# Treatment patterns in children with autism in the United States

**DOI:** 10.1002/aur.2070

**Published:** 2019-01-10

**Authors:** Brigitta U. Monz, Richard Houghton, Kiely Law, Georg Loss

**Affiliations:** ^1^ Personalized Health Care Data Science, Real World Data F. Hoffmann‐La Roche Ltd. Basel Switzerland; ^2^ Department of Clinical Pharmacy and Toxicology, School CAPHRI Maastricht UMC+ Maastricht The Netherlands; ^3^ Kennedy Krieger Institute Interactive Autism Network Baltimore Maryland; ^4^ Department of Pediatrics Johns Hopkins University School of Medicine Baltimore Maryland

**Keywords:** autism spectrum disorder, children, service use, access to care, urban, rural, Medicaid, private insurance

## Abstract

Children with autism receive different types of non‐drug treatments. We aimed to describe caregiver‐reported pattern of care and its variability by geography and healthcare coverage in a US‐wide sample of children aged 3–17 years. We recruited caregivers from the Simons Foundation Powering Autism Research for Knowledge (SPARK) cohort. Two online questionnaires (non‐drug treatment, Autism Impact Measure) were completed in September/October 2017. Primary outcome measures were caregiver‐reported types and intensities of treatments (behavioral, developmental/relationship, speech and language (SLT), occupational, psychological, “other”; parent/caregiver training) in the previous 12 months. Main explanatory variables were geography and type of healthcare coverage. We investigated associations between the type/intensity of treatments and geography (metropolitan/nonmetropolitan) or coverage (Medicaid vs privately insured by employer) using regression analysis. Caregivers (*n* = 5,122) were mainly mothers (92.1%) with mean (*SD*) age of 39.0 (7.3) years. Mean child age was 9.1 (3.9) years; mostly males (80.0%). Almost all children received at least one intervention (96.0%). Eighty percent received SLT or occupational therapy, while 52.0% received both. Behavioral therapy and SLT were significantly more frequent and more intense in metropolitan than in nonmetropolitan areas. No consistently significant associations were seen between healthcare coverage and frequency or intensity of interventions. At least one barrier such as “waiting list” and “no coverage” was reported by 44.8%. In conclusion, in children sampled from SPARK, we observed differences between metropolitan and nonmetropolitan areas, while we did not find significant differences between those privately insured versus Medicaid. ***Autism Res*** 2019, 12: 517–526 © 2019 The Authors. *Autism Research published by International Society for Autism Research* published by Wiley Periodicals, Inc.

**Lay Summary:**

The American Academy of Child and Adolescent Psychiatry recommends the use of multiple treatment modalities in autism spectrum disorder (ASD). We wanted to understand what types of treatment children (aged 3–17 years) with ASD receive in the United States, how and where the treatments take place and for how long. We invited caregivers from Simons Foundation Powering Autism Research for Knowledge (“SPARK ,” https://sparkforautism.org/) to complete the study questions online. Participants reported on utilization of conventional, non‐drug treatments for ASD, including behavioral interventions, developmental/relationship interventions, speech and language therapy (SLT), occupational therapy, psychological therapy, and parent/caregiver training. People that completed the study (*n* = 5,122) were primarily mothers of the child with ASD (92%); most of the children were boys (80%). The ASD care for the child was mostly coordinating by the mother. Almost all children received at least some type of non‐drug therapies (96%), most often SLT and/or occupational therapy, mainly provided in school. Behavioral therapy was most often received in public school in rural areas, while at home in urban areas. We saw less use of behavioral therapy and SLT in rural areas, but overall comparable use between children covered by Medicaid and those covered by private insurance. Almost half the caregivers reported at least one barrier to treatment, such as “waiting list” and “no coverage.” More than half said that their child benefited “much” or “very much” from the therapies received. While overall non‐drug treatment rates for children with ASD were high in the United States in our study, differences existed depending on where the family lives; not only regarding the type of therapy, but also where it takes place.

## Introduction

Autism spectrum disorder (ASD) is a family of neurodevelopmental disorders characterized by repetitive or stereotyped behaviors and deficits in social interactions. An estimated 11.9 in 1,000 children in the United States have ASD [Durkin et al., 2017]. The American Academy of Child and Adolescent Psychiatry recommends the use of multiple treatment modalities in ASD [2014]. Conventional non‐drug treatments, including behavioral interventions, speech and language therapy (SLT), and occupational therapy, are utilized across pediatric age groups and administered in diverse settings (e.g., home, school, and specialty clinics/offices) [Nguyen, Krakowiak, Hansen, Hertz‐Picciotto, & Angkustsiri, 2016]. Previous studies reporting on the pattern of care received by children with ASD either relied on a network of centers or providers [e.g., Becerra et al., 2017], utilized subsections of existing U.S. surveys [e.g., Vohra, Madhavan, Sambamoorthi, & St Peter, 2014], used claims analysis [e.g., Candon et al., 2018], or investigated certain age groups [e.g., Zuckerman, Lindly, & Chavez, 2017; Payakachat, Tilford, & Kuhlthau, 2018].

Prior research has raised concerns that children in more rural settings have access to fewer services [Kelleher & Gardner, 2017]; as well as that the type of healthcare coverage may dictate utilization of services, specifically that Medicaid provided for more interventions than commercial plans [Wang, Mandell, Lawer, Cidav, & Leslie, 2013]. Therefore, the goal of our study was to describe the caregiver‐reported pattern of non‐drug ASD treatment and its variation by geographic region and type of healthcare coverage across the United States in children aged 3–17 years. Our study recruited caregivers from the Simons Foundation Powering Autism Research for Knowledge (SPARK) cohort, a U.S.‐based online research cohort with individuals and families who have consented to providing information and medical samples to further autism research [SPARK Consortium, 2018].

## Methods

Online surveys for non‐drug therapy and for the Autism Impact Measure (AIM; to assess frequency and impact of ASD symptoms [Kanne et al., 2014; Mazurek et al., 2018]) were sent to caregivers (i.e., parents and guardians/legally authorized representatives) in the SPARK cohort between September 13 and October 22, 2017. Invitations were sent in four batches, first inviting potentially eligible caregivers (i.e., have children with ASD registered in SPARK in required age range) that had most recently joined SPARK. Participants and their oldest ASD dependent aged 3–17 years (hereafter “child[ren] with ASD”) had to have been living in the same household, with the caregiver as the main person supporting this child for at least the preceding 12 months. SPARK currently provides information in English and requires for inclusion that participants be able to read and understand English. The research protocol was approved by an institutional review board (Western IRB) and participants consented online. Upon completion of both surveys, participants received a $20 online shop voucher via email.

The primary outcome measures were the types and intensities of non‐drug treatments in the preceding 12 months as reported by the caregiver, categorized into seven groups (child‐directed: behavioral, developmental and/or relationship‐based, SLT, occupational, psychological, and “other”; and parent/caregiver training). This categorization was similar to Salomone et al. [2016] in order to allow comparisons, with the only difference being that we separated out psychological interventions from the “other” category. The main explanatory variables were geography of residence and healthcare coverage type. Geography was defined by a six‐level urban–rural classification scheme based on U.S. state and county (i.e., six‐level metropolitan statistical area [MSA] [Ingram & Franco, 2014]). For the analysis of the association between geography and outcomes, we collapsed this to two levels (i.e., two‐level MSA), nonmetropolitan and metropolitan, indicating “rural” and “urban,” as per the classification scheme. Healthcare coverage types were categorized into mutually exclusive groups: those with only Medicaid (“Medicaid‐only”), those with only private insurance provided by an employer (“private insurance‐only”), one other type of coverage, more than one, and uninsured. Barriers [adapted from Chiri & Warfield, 2012], caregiver's role in access to and perceived benefits of non‐drug treatments, demographic characteristics of caregivers and children with ASD, and AIM scores were also analyzed.

### *Statistical Methods*

Data were summarized descriptively. Types and intensities of non‐drug treatments were also stratified by geography, healthcare coverage type, and age group.

To model the associations between explanatory variables (geography, type of healthcare coverage) and outcomes, we identified covariates needed for adjustment using directed acyclic graphs [Greenland, Pearl, & Robins, 1999, Supporting Information Figs. S1 and S2], followed by propensity score methodology (inverse probability weighting) to create balance in the covariates, and finally applied regression modeling (logistic for treatment types; negative binomial for intensities). Populations did not sufficiently overlap to allow modeling the association between all four categories of insurance (i.e., Medicaid, private provided by employer, one type of coverage from the other categories, more than one coverage type) simultaneously, using multinomial logistic regression to derive propensity scores. We therefore present only a comparison between private via employer versus Medicaid. For further details, see Supporting Information Methods and Supporting Information Tables.

An intervention was counted as “absent” if a response of “don't know” was given. For present treatments, missing intensity values were set to 0.5 hr/week. These imputations were necessary <5% of the time as the data were generally very well populated. For AIM, no total or domain scores were calculated if >20% of items had missing responses.

We justified the sample size target of 5,000 based on the following: if 85% of children received at least one non‐drug treatment and up to 16 strata analyzed, a precision of 0.85 (95% confidence interval [CI]: 0.81, 0.89) could be achieved, which was considered adequate.

R version 3.3.2 was used for all analyses.

## Results

Invitations were emailed to 11,514 of 19,142 potentially eligible caregivers. The non‐drug treatment survey was completed by 5,122 (44.5% of those invited), and the AIM by 5,001 (43.4%; Supporting Information Fig. S3). The study was closed online when the targeted sample size had been reached.

### *Characteristics of Caregivers and Children with ASD*

The majority (92.1%) of the caregivers were mothers, with a mean age of 39.0 years and were mostly (76.5%) White/non‐Hispanic (Table 1). Two‐thirds had a higher education (completed college or higher) and most (81.2%) lived in metropolitan areas. The children with ASD were predominantly (80.0%) male, with a mean age of 9.1 years, and mostly (68.5%) White/non‐Hispanic. About two‐thirds had been diagnosed before age 5. Almost all had at least some insurance coverage. Of those who reported having ever had an IQ test, 44.6% scored <100.

**Table 1:** Characteristics of Caregivers and Their Children with ASD (*n* = 5,122)

Characteristic	Number (%)
	[except where indicated otherwise]
*Caregivers*	
Age, years [mean (*SD*)]	39.02 (7.30)
Relation to child	
Mother	4719 (92.1)
Father	314 (6.1)
Legal guardian	55 (1.1)
Other	34 (0.7)
Married/living with partner	4101 (80.1)
Completed college or higher	3259 (63.6)
Employment	
Working (full or part‐time)	3024 (59.0)
Full‐time homemaker	1541 (30.1)
Other	557 (10.9
Race/ethnicity	
White/non‐Hispanic	3919 (76.5)
White/Hispanic	301 (5.9)
Non‐white/non‐Hispanic	650 (12.7)
Non‐white/Hispanic	252 (4.9)
More than one child with autism in family	801 (15.6)
Region	
West	1297 (25.3)
Midwest	1124 (21.9)
Northeast	825 (16.1)
South	1868 (36.5)
Unknown	8 (0.2)
Metropolitan statistical area	
Metropolitan	4158 (81.2)
Nonmetropolitan	588 (11.5)
Unknown	376 (7.3)
Self‐reported health	
Excellent	838 (16.4)
Very good	2049 (40.0)
Good	1699 (33.2)
Fair	448 (8.7)
Poor	82 (1.6)
Missing	6 (0.1)
Household income	
<$20,000	512 (10.0)
$20,000–$34,999	696 (13.6)
$35,000–$49,999	619 (12.1)
$50,000–$74,999	904 (17.6)
$75,000–$99,999	681 (13.3)
$100,000–$124,999	577 (11.3)
$125,000–$149,999	312 (6.1)
$150,000 or more	576 (11.2)
Missing	245 (4.8)
*Children with autism (age 3 to <18)*	
Age, years [mean (*SD*)]	9.10 (3.92)
Male	4096 (80.0)
Race/ethnicity	
White/non‐Hispanic	3510 (68.5)
White/Hispanic	501 (9.8)
Non‐white/non‐Hispanic	830 (16.2)
Non‐white/Hispanic	281 (5.5)
Caregiver‐reported child's health	
Excellent	1694 (33.1)
Very good	2174 (42.4)
Good	1063 (20.8)
Fair	169 (3.3)
Poor	13 (0.3)
Missing	9 (0.2)
Autism diagnosis	
ASD	3786 (73.9)
Autism/autistic disorder	553 (10.8)
Asperger syndrome	455 (8.9)
PDDNOS	289 (5.6)
Unknown/missing	39 (0.8)
Age at autism diagnosis, years	
0–2	1337 (26.1)
3–4	1999 (39.0)
5–9	1418 (27.7)
>9	348 (6.8)
Missing	20 (0.4)
Years since autism diagnosis	
0–1	1093 (21.3)
2–3	1303 (25.4)
4–5	883 (17.2)
6–9	1130 (22.1)
≥10	697 (13.6)
Missing	16 (0.3)
Insurance	
Uninsured/unknown	87 (1.7)
Only Medicaid	1564 (30.5)
Only private (via employer)	2083 (40.7)
One type of other coverage	418 (8.2)
More than one type	970 (18.9)
Insurance drug coverage	4672 (91.2)
Prescription drug use	
Overall	2683 (52.4)
Drugs for autism	1718 (33.5)
Over‐the‐counter drug use	
Overall	3106 (60.6)
Drugs for autism	965 (18.8)
Medical problems	2348 (45.8)
Other mental health or psychiatric problems	2424 (47.3)
Primary care physician main healthcare provider	2911 (56.8)
IQ test results^a^	
≤70	464 (20.1)
71–99	564 (24.5)
≥100	789 (34.2)
Unknown	488 (21.2)
Attending school with special education students only	1109 (21.7)
Spending more than 60% of classroom time with typically developing peers	2312 (45.1)
	

ASD: Autism Spectrum Disorder; IQ: intelligence quotient; PDDNOS: pervasive developmental disorder – not otherwise specified; *SD*: standard deviation.

a IQ test results were available for *n* = 2,305 (45.0%).

There were a few demographic differences between children enrolled in special‐education‐only schools (21.7% of the total) and the overall group; notably, mean age was lower, a lower proportion were of White/non‐Hispanic ethnicity, a higher proportion had been diagnosed before 5 years, and had IQ test scores of 70 or below (Supporting Information Table S1).

The mean (*SD*) total AIM score was 220.8 (54.1); possible score range: 82–410, with higher scores indicating higher symptom frequency/impact. Mean (*SD*) [possible range] domain scores were: repetitive behavior, 41.1 (13.8) [16–80]; communication, 30.6 (11.9) [12–60]; atypical behavior, 34.7 (10.1) [12–60]; social reciprocity 27.1 (7.4) [10–50]; and peer interaction, 22.9 (7.1) [8–40].

### *Types of Treatments*

As shown in Table 2, 96.0% of children received at least one type of non‐drug treatment, the most common being SLT (71.4%). A higher proportion of children in metropolitan versus nonmetropolitan areas received behavioral therapy (57.2% vs 46.4%) and SLT (72.3% vs 65.0%). There was a pattern toward lower utilization across urbanization categories from large central metro to noncore for behavioral therapy, SLT, and parent/caregiver training (Supporting Information Table S2). Similar proportions of children covered by Medicaid‐only and private insurance‐only received at least one therapy (96.2% vs 95.4%); although children under Medicaid‐only received occupational therapy more frequently (61.8% vs 55.7%) and “other” therapies less frequently (63.4% vs 69.9%). For most therapies, use decreased from the lowest age group to the highest age group.

**Table 2:** Caregiver‐Reported Types of Non‐Drug Treatments in the Last 12 Months Provided to Children with ASD (*n* = 5,122)

	*N*	Any	Behavioral	Developmental/relationship	SLT	Occupational	Psychological	Other	Parent/caregiver training
Overall	5,122	4,918 (96.0)	2,875 (56.1)	1,348 (26.3)	3,657 (71.4)	3,078 (60.1)	1,471 (28.7)	3,471 (67.8)	1,515 (29.6)
Geography									
Metropolitan	4,158	4,001 (96.2)	2,378 (57.2)	1,081 (26.0)	3,005 (72.3)	2,500 (60.1)	1,210 (29.1)	2,828 (68.0)	1,253 (30.1)
Nonmetropolitan	588	551 (93.7)	273 (46.4)	152 (25.9)	382 (65.0)	334 (56.8)	157 (26.7)	377 (64.1)	142 (24.1)
Unknown	376	366 (97.3)	224 (59.6)	115 (30.6)	270 (71.8)	244 (64.9)	104 (27.7)	266 (70.7)	120 (31.9)
Insurance									
Uninsured/unknown	87	75 (86.2)	43 (49.4)	23 (26.4)	55 (63.2)	44 (50.6)	17 (19.5)	47 (54.0)	16 (18.4)
Only Medicaid	1,564	1,505 (96.2)	860 (55.0)	405 (25.9)	1,129 (72.2)	967 (61.8)	469 (30.0)	992 (63.4)	401 (25.6)
Only private (via employer)	2,083	1,987 (95.4)	1,103 (53.0)	542 (26.0)	1,429 (68.6)	1,160 (55.7)	607 (29.1)	1,455 (69.9)	620 (29.8)
One type of other coverage	418	399 (95.5)	246 (58.9)	112 (26.8)	268 (64.1)	223 (53.3)	116 (27.8)	270 (64.6)	129 (30.9)
More than one type	970	952 (98.1)	623 (64.2)	266 (27.4)	776 (80.0)	684 (70.5)	262 (27.0)	707 (72.9)	349 (36.0)
Child age (years)^a^									
3–4	690	679 (98.4)	452 (65.5)	238 (34.5)	626 (90.7)	525 (76.1)	77 (11.2)	381 (55.2)	253 (36.7)
5–9	2,183	2,122 (97.2)	1,304 (59.7)	541 (24.8)	1,770 (81.1)	1,572 (72.0)	463 (21.2)	1,464 (67.1)	695 (31.8)
10–14	1,641	1,555 (94.8)	836 (50.9)	432 (26.3)	970 (59.1)	792 (48.3)	672 (41.0)	1,205 (73.4)	415 (25.3)
15–17	597	551 (92.3)	274 (45.9)	134 (22.4)	283 (47.4)	179 (30.0)	256 (42.9)	413 (69.2)	148 (24.8)

ASD: Autism Spectrum Disorder; SLT: speech and language therapy.

Numbers represent *n* (%) that were treated in each subgroup.

a Missing age, *n* = 11.

Most caregivers reported therapy as ongoing (i.e., not having ended in the 12‐month recall period; ranging from 61.0% for parent/caregiver training to 89.1% for SLT). Approximately 42% reported four or more interventions. Children were most likely to receive SLT and occupational therapy concurrently; followed by parent/caregiver training and behavioral therapy; Supporting Information Table S3. The most common interventions used concurrently were behavioral therapy/SLT/occupational therapy/“other” (7.1%) and SLT/occupational therapy/“other” (5.9%); Supporting Information Table S4. In nonmetropolitan areas, behavioral therapy was not in the three most common concurrently used interventions, and developmental/relationship‐based and parental training not in any of the combinations occurring for >2% of the children. Overall, 52.0% received at least SLT and occupational therapy, while 79.5% received at least either.

### *Intensity of Treatments*

The median intensity of all treatments was 6.0 hr/week, with behavioral therapy being the most intense (4.0 hr/week, Table 3). Metropolitan areas reported higher intensity for “any” therapy, behavioral therapy, “other” therapy, and developmental/relationship‐based interventions (also Supporting Information Fig. S4 and Table S5). A difference between Medicaid‐only and private insurance‐only patients was seen only for behavioral therapy (2.0 and 4.0 hr/week, respectively) and “other” (also Supporting Information Fig. S5). Although there was no consistent pattern overall across age groups, the two lowest (3–4 and 5–9 years) had the highest intensity for behavioral therapy.

**Table 3:** Median Intensity^a^ and Setting of Non‐Drug Treatments in the Last 12 Months Provided to Children with ASD (*n* = 4,918)

	*N* (any)	All	Behavioral			Developmental/relationship			SLT			Occupational			Psychological			Other		
		Int	Int	IGR	SNR	Int	IGR	SNR	Int	IGR	SNR	Int	IGR	SNR	Int	IGR	SNR	Int	IGR	SNR
Overall	4,918	6.0	4.0	2.16	0.64	2.0	1.35	0.99	1.0	1.74	1.79	1.0	2.93	1.33	1.0	3.27	0.40	2.0	1.21	0.99
Geography																				
Metropolitan	4,001	6.0	4.0	2.09	0.65	2.0	1.30	1.00	1.0	1.67	1.79	1.0	2.82	1.31	1.0	3.11	0.41	2.0	1.16	0.99
Nonmetropolitan	551	4.5	2.0	3.21	0.56	1.0	1.67	0.98	1.0	2.46	1.89	1.0	3.73	1.59	1.0	4.72	0.36	1.0	1.60	1.03
Unknown	366	5.5	3.0	2.04	0.66	2.0	1.48	0.90	1.0	1.81	1.61	1.0	3.28	1.29	1.0	3.72	0.39	1.0	1.28	0.98
Insurance																				
Uninsured	75	4.5	3.0	1.65	1.04	1.0	1.27	1.80	1.0	2.09	2.83	1.0	2.53	1.89	1.0	3.00	0.89	1.0	1.28	1.35
Only Medicaid	1,505	5.5	2.0	2.62	0.66	2.0	1.60	0.97	1.0	2.20	1.70	1.0	3.54	1.30	1.0	3.34	0.43	2.0	1.45	1.03
Only private (via employer)	1,987	5.5	4.0	1.88	0.68	2.0	1.18	1.05	1.0	1.46	2.04	1.0	2.52	1.40	1.0	3.14	0.41	1.0	1.07	1.01
One type of other coverage	399	6.5	5.5	2.47	0.46	2.0	1.47	0.90	1.0	1.86	1.41	1.0	3.22	0.99	1.0	5.50	0.34	1.0	1.32	0.89
More than one type	952	8.0	5.0	2.12	0.62	2.0	1.36	0.91	1.0	1.73	1.60	1.0	2.97	1.38	1.0	2.91	0.36	2.0	1.16	0.92
Child age (years)^b^																				
3–4	679	10.0	9.0	2.39	0.66	3.0	1.82	0.85	1.0	2.61	1.05	1.0	3.83	0.86	1.0	2.36	0.43	1.0	1.75	0.77
5–9	2,122	7.0	5.0	2.10	0.65	2.0	1.36	1.02	1.0	1.78	1.71	1.0	3.03	1.34	1.0	2.74	0.49	1.0	1.25	0.95
10–14	1,555	5.0	2.0	2.13	0.65	2.0	1.23	1.01	1.0	1.44	2.72	1.0	2.47	1.85	1.0	3.70	0.38	2.0	1.10	1.10
15–17	551	4.5	2.0	2.22	0.57	1.0	1.09	1.07	1.0	1.31	3.12	1.0	2.22	1.51	1.0	3.79	0.31	2.0	1.02	1.06

ASD: Autism Spectrum Disorder; Int: intensity (median hours/week), IGR: individual to group sessions ratio, SLT: speech and language therapy; SNR: in school to not in school sessions ratio.

a Expressed as median hr/week.

b Missing age, *n* = 11.

### *Setting of Treatments*

Non‐drug therapies were more often given in individual rather than group sessions, with psychological interventions and occupational therapy having the highest individual‐to‐group ratios (IGRs: 3.27 and 2.93, respectively, Table 3). Children in nonmetropolitan areas were more likely to receive individual sessions than those in metropolitan areas; with notable differences seen for behavioral therapy, psychological interventions, occupational therapy, and SLT. Children under Medicaid‐only more often received individual sessions than those under private insurance‐only; with differences seen for occupational therapy, SLT, and behavioral therapy. There was a pattern of individual sessions from lowest in large central metro to highest in noncore (Supporting Information Table S5).

SLT and occupational therapy were more often provided in school (school/not in school ratio [SNR]: 1.79 and 1.33, respectively), while behavioral therapy and psychological interventions were more frequently provided outside school (SNR: 0.64 and 0.40, respectively). There were no notable differences in SNR's between metropolitan and nonmetropolitan areas. A small difference was seen between the Medicaid‐only and private insurance‐only patients for SLT.

The most common place of care was the home for behavioral interventions (45.0%); public school for developmental/relationship‐based interventions (56.0%), SLT (76.5%), occupational therapy (63.6%), and “other” interventions (57.7%); and private therapist (57.8%) for psychological interventions (Table 4). Behavioral therapy was most often received in public school in nonmetropolitan areas (44.7%), but at home in metropolitan areas (46.3%).

**Table 4:** Place of Non‐Drug ASD Therapy ‐ by Intervention Type

Variable	*n* (%)					
	Behavioral	D/R	SLT	OT	Psychological	Other
Overall (*N*)	2,875	1,348	3,657	3,078	1,471	3,471
Home	1,295 (45.0)	403 (29.9)	423 (11.6)	354 (11.5)	182 (12.4)	996 (28.7)
Public school or pre‐school	1,214 (42.2)	755 (56.0)	2,798 (76.5)	1,958 (63.6)	406 (27.6)	2,004 (57.7)
Private school or pre‐school	351 (12.2)	152 (11.3)	364 (10.0)	294 (9.6)	93 (6.3)	356 (10.3)
OP medical/autism clinic	535 (18.6)	195 (14.5)	407 (11.1)	469 (15.2)	271 (18.4)	435 (12.5)
Private therapist/clinician office	900 (31.3)	391 (29.0)	1,003 (27.4)	885 (28.8)	850 (57.8)	866 (24.9)
Daycare or before/after school program	90 (3.1)	29 (2.2)	33 (0.9)	25 (0.8)	6 (0.4)	78 (2.2)
Camp	183 (6.4)	72 (5.3)	43 (1.2)	33 (1.1)	21 (1.4)	452 (13.0)
Other location	152 (5.3)	82 (6.1)	43 (1.2)	52 (1.7)	94 (6.4)	341 (9.8)
Unknown location	3 (0.1)	4 (0.3)	5 (0.1)	3 (0.1)	3 (0.2)	61 (1.8)

ASD: Autism Spectrum Disorder; D/R: Developmental and/or relationship‐based intervention; OP: Outpatient OT: Occupational Therapy; SLT: Speech and Language Therapy.

Note: Place of non‐drug therapy is not applicable for “Any” intervention; and was not collected for Parent/Caregiver Training intervention.

### *Barriers to Treatments*

Overall, 44.8% reported at least one barrier to non‐drug therapy. “Waiting list” (26.4%) was the most common provider‐related barrier (Table 5), whereas “no coverage” (17.9%) and “cost” (16.7%) were the most common health‐plan‐related barriers (Table 6). Metropolitan areas reported a higher frequency of “waiting list” than nonmetropolitan areas, but a much lower frequency of “not available in area” (15.1% vs 32.0%).

**Table 5:** Provider Barriers to Non‐Drug Therapy

Subgroup		*N*	Provider barrier *n* (%)							
			Any	Not available in area	Transportation	Timing	Waiting list	Provider did not know how to treat	Dissatisfaction with provider	Did not know where to go
Overall		5,122	2,296 (44.8)	881 (17.2)	265 (5.2)	576 (11.2)	1,350 (26.4)	503 (9.8)	512 (10.0)	605 (11.8)
6‐level MSA	Large central metro	1,248	572 (45.8)	138 (11.1)	63 (5.0)	169 (13.5)	369 (29.6)	106 (8.5)	134 (10.7)	134 (10.7)
	Large fringe metro	1,527	682 (44.7)	236 (15.5)	75 (4.9)	183 (12.0)	408 (26.7)	149 (9.8)	166 (10.9)	179 (11.7)
	Medium metro	938	401 (42.8)	151 (16.1)	59 (6.3)	87 (9.3)	232 (24.7)	92 (9.8)	91 (9.7)	116 (12.4)
	Small metro	445	202 (45.4)	104 (23.4)	18 (4.0)	46 (10.3)	116 (26.1)	46 (10.3)	43 (9.7)	59 (13.3)
	Nonmetropolitan: micropolitan	369	157 (42.5)	102 (27.6)	17 (4.6)	31 (8.4)	77 (20.9)	47 (12.7)	30 (8.1)	44 (11.9)
	Nonmetropolitan: noncore	219	105 (47.9)	86 (39.3)	19 (8.7)	22 (10.0)	44 (20.1)	27 (12.3)	17 (7.8)	31 (14.2)
	Unknown	376	177 (47.1)	64 (17.0)	14 (3.7)	38 (10.1)	104 (27.7)	36 (9.6)	31 (8.2)	42 (11.2)
2‐level MSA	Metropolitan	4,158	1,857 (44.7)	629 (15.1)	215 (5.2)	485 (11.7)	1,125 (27.1)	393 (9.5)	434 (10.4)	488 (11.7)
	Nonmetropolitan	588	262 (44.6)	188 (32.0)	36 (6.1)	53 (9.0)	121 (20.6)	74 (12.6)	47 (8.0)	75 (12.8)
	Unknown	376	177 (47.1)	64 (17.0)	14 (3.7)	38 (10.1)	104 (27.7)	36 (9.6)	31 (8.2)	42 (11.2)
Healthcare coverage	Uninsured (including do not know)	87	44 (50.6)	17 (19.5)	5 (5.7)	7 (8.0)	22 (25.3)	9 (10.3)	6 (6.9)	13 (14.9)
	Medicaid only	1,564	713 (45.6)	315 (20.1)	122 (7.8)	158 (10.1)	431 (27.6)	190 (12.1)	175 (11.2)	217 (13.9)
	Private (via employer) only	2,083	873 (41.9)	290 (13.9)	71 (3.4)	235 (11.3)	488 (23.4)	162 (7.8)	166 (8.0)	197 (9.5)
	One type of other coverage	418	211 (50.5)	81 (19.4)	17 (4.1)	46 (11.0)	121 (28.9)	50 (12.0)	43 (10.3)	54 (12.9)
	More than one type	970	455 (46.9)	178 (18.4)	50 (5.2)	130 (13.4)	288 (29.7)	92 (9.5)	122 (12.6)	124 (12.8)

MSA: Metropolitan Statistical Area.

**Table 6:** Health Plan and Other Barriers to Non‐Drug Therapy

Subgroup		*N*	Health plan and other barriers *n* (%)						
			Any	Cost	No insurance	No coverage	No referral	Provider does not accept insurance	Other
Overall		5,122	2,296 (44.8)	854 (16.7)	64 (1.2)	918 (17.9)	179 (3.5)	692 (13.5)	315 (6.1)
Six‐level MSA	Large central metro	1,248	572 (45.8)	242 (19.4)	19 (1.5)	245 (19.6)	54 (4.3)	196 (15.7)	75 (6.0)
	Large fringe metro	1,527	682 (44.7)	288 (18.9)	22 (1.4)	297 (19.4)	45 (2.9)	239 (15.7)	98 (6.4)
	Medium metro	938	401 (42.8)	128 (13.6)	9 (1.0)	157 (16.7)	30 (3.2)	107 (11.4)	52 (5.5)
	Small metro	445	202 (45.4)	68 (15.3)	2 (0.4)	68 (15.3)	18 (4.0)	52 (11.7)	27 (6.1)
	Nonmetropolitan: micropolitan	369	157 (42.5)	51 (13.8)	5 (1.4)	50 (13.6)	14 (3.8)	35 (9.5)	24 (6.5)
	Nonmetropolitan: noncore	219	105 (47.9)	21 (9.6)	1 (0.5)	31 (14.2)	8 (3.7)	17 (7.8)	15 (6.8)
	Unknown	376	177 (47.1)	56 (14.9)	6 (1.6)	70 (18.6)	10 (2.7)	46 (12.2)	24 (6.4)
Two‐level MSA	Metropolitan	4,158	1,857 (44.7)	726 (17.5)	52 (1.3)	767 (18.4)	147 (3.5)	594 (14.3)	252 (6.1)
	Nonmetropolitan	588	262 (44.6)	72 (12.2)	6 (1.0)	81 (13.8)	22 (3.7)	52 (8.8)	39 (6.6)
	Unknown	376	177 (47.1)	56 (14.9)	6 (1.6)	70 (18.6)	10 (2.7)	46 (12.2)	24 (6.4)
Healthcare coverage	Uninsured (including do not know)	87	44 (50.6)	24 (27.6)	20 (23.0)	18 (20.7)	3 (3.4)	14 (16.1)	4 (4.6)
	Medicaid only	1,564	713 (45.6)	195 (12.5)	13 (0.8)	249 (15.9)	76 (4.9)	217 (13.9)	103 (6.6)
	Private (via employer) only	2,083	873 (41.9)	391 (18.8)	12 (0.6)	375 (18.0)	35 (1.7)	251 (12.0)	115 (5.5)
	One type of other coverage	418	211 (50.5)	56 (13.4)	7 (1.7)	70 (16.7)	32 (7.7)	48 (11.5)	30 (7.2)
	More than one type	970	455 (46.9)	188 (19.4)	12 (1.2)	206 (21.2)	33 (3.4)	162 (16.7)	63 (6.5)

MSA: Metropolitan Statistical Area.

### *Caregiver's Role and Perception*

Table 7 lists the non‐drug therapies in the “other” category. The most frequent therapies were social skills training (37.0%) and academic support (28.3%). The main care coordinator was the caregiver (81.9%). Three quarters reported that they were satisfied with the current level of care, and 58.2% reported that their child benefited “much” or “very much” from care.

**Table 7:** Non‐Drug ASD Therapies in the “Other” Category (*n* = 3,471)

Type	*n*	%
Social skills training	1,894	36.98
Academic support (for example reading, writing, and math tutor)	1,450	28.31
Sensory integration	792	15.46
Physical therapy	739	14.43
Recreational^a^	715	13.96
Biomedical^b^	431	8.41
Animal‐assisted activities and therapies^c^	395	7.71
Other therapy not previously mentioned	395	7.71
Other therapy – but do not know which	234	4.57
Fast Forward, APE	206	4.02
Structured teaching (TEACCH)	177	3.46
SCERTS	152	2.97
AIT	78	1.52
The Built Environment	2	0.04

AIT: auditory integration training/therapy; APE: Adaptive physical education; ASD: Autism Spectrum Disorder; SCERTS: Social Communication/Emotional Regulation/Transactional Support; TEACCH: Training and Education of Autistic and Related Communication Handicapped Children.

a For example, specialized summer camp.

b For example, biofeedback, special diets, vitamins.

c For example, therapies including dogs, horses, dolphins.

### *Associations between Treatments and Geography/Insurance*

As shown in Table 8, the odds of receiving “any” treatment, behavioral therapy and SLT were significantly greater in metropolitan areas than in nonmetropolitan areas (odds ratio [OR] 1.71, 1.54, and 1.41, respectively). No consistently (i.e., significant in both, the PS‐adjusted and double‐adjusted analyses) significant associations were seen between type of insurance (Medicaid‐only vs private insurance‐only) and type of treatment received.

**Table 8:** Association of Caregiver‐Reported Type and Intensity of Non‐Drug Therapy with Geography and Insurance

Non‐drug therapy type	Geography (metropolitan vs nonmetropolitan)	Insurance^a^, ^b^	
		(Only private via employer vs only Medicaid)	
	*N* = 4,746	*N* = 1,632	
	Unadjusted	PS‐adjusted	Double‐adjusted
*Association with type of therapy: OR (95% CI)*			
Any	1.71 (1.17,2.45)	0.63 (0.34,1.17)	0.58 (0.32,1.06)
Behavioral	1.54 (1.30,1.83)	1.12 (0.78,1.62)	1.07 (0.78,1.47)
Developmental/ relationship	1.01 (0.83,1.23)	0.99 (0.65,1.52)	1.04 (0.73,1.50)
SLT	1.41 (1.17,1.69)	0.67 (0.44,1.03)	0.66 (0.45,0.97)
Occupational	1.15 (0.96,1.36)	0.80 (0.54,1.19)	0.77 (0.55,1.09)
Psychological	1.13 (0.93,1.37)	0.77 (0.55,1.09)	0.69 (0.49,0.97)
Other	1.19 (0.99,1.42)	0.86 (0.58,1.26)	0.86 (0.62,1.20)
*Association with intensity of therapy: RR (95% CI)*			
Any	1.35 (1.23,1.48)	0.90 (0.75,1.07)	0.84 (0.72,0.97)
Behavioral	1.71 (1.45,2.01)	1.02 (0.81,1.27)	0.98 (0.78,1.24)
Developmental/relationship	1.23 (0.95,1.57)	1.37 (0.93,2.01)	0.99 (0.70,1.39)
SLT	1.18 (1.06,1.31)	0.90 (0.70,1.17)	0.88 (0.72,1.07)
Occupational	1.04 (0.93,1.17)	0.83 (0.64,1.08)	0.83 (0.66,1.05)
Psychological	1.46 (1.19,1.79)	0.89 (0.60,1.32)	0.81 (0.56,1.17)
Other	1.19 (1.04,1.35)	0.66 (0.54,0.81)	0.65 (0.54,0.80)

CI: Confidence interval; OR: Odds Ratio; PS: propensity score; RR: Rate ratio; SLT: Speech and language therapy.

Variables for adjustment: AIM domain scores (continuous), child race/ethnicity (White/Hispanic, White/Non‐Hispanic, Non‐White/Hispanic, Non‐White/Non‐Hispanic), child other medical problems (yes/no), child other mental health or psychiatric problems (yes/no), geography (nonmetropolitan/ metropolitan), household income (four strata, ≥$20,000 to ≤$99,999), marital status (married/living with partner yes/no), mother employment (work full‐time/part time yes/no), US state (excluded states with *n* < 3 for Medicaid and private employer insurance).

a Models for insurance were adjusted for below variables using propensity score inverse probability weighting (adjusted and double‐adjusted: see Methods section and Supplementary Appendix).

b See Supporting Information Table S6 for trimmed results.

The intensity of “any” treatment was significantly greater in metropolitan areas than in nonmetropolitan areas (relative risk, 1.35), based on the intensities of behavioral therapy, SLT, psychological and “other” interventions that were all significantly greater in metropolitan areas. The RRs for the intensity of any of the treatment options did not indicate a consistently significant difference between the two types of healthcare coverage, except for “other,” where the rates were lower for those with private insurance provided by employer. For results on the six‐level MSA and other additional analyses, refer to the Supporting Information Tables S6–S9.

## Discussion

This study investigated the caregiver‐reported pattern of non‐drug therapy and the variability of care by geographic region and type of healthcare coverage, in children with ASD.

The cohort was largely representative of children with ASD in the United States. U.S. population weighted survey data, from the 2016 National Survey of Children's Health [Data Resource Center for Child & Adolescent Health, 2018], reported a similar male‐to‐female ratio (around 80% male), use of medications for autism (around 1/3), age at diagnosis (around 1/3 beyond age 5), and insurance coverage (around 98% with coverage). The sample of the 2016 survey included close to 70% White/non‐Hispanics, as in our study, while the population estimate was around 53%, indicating over‐representation of this group among the survey respondents.

Salomone et al. [2016] who grouped therapies similarly, reported that 91% of respondents from 18 European countries used at least one non‐drug therapy (vs 96% in our study). Behavioral therapy, SLT, and occupational therapy were reported at 32%, 64%, and 35%, although there was wide variation across countries. Our study reported higher rates [66% and 60%, 91% and 81%, 76% and 72% for these therapies, for the age groups 3–4 and 5–9 years, respectively, corresponding best to the age range investigated by Salomone et al., 2016]. Most children in our cohort received more than one non‐drug therapy. Approximately one‐quarter used three therapies concurrently and two‐thirds used three or more in the past 12 months; the most common combination being behavioral therapy/SLT/occupational therapy/“other” (7.1%). It is difficult to compare these rates with other studies as they either did not use similar groupings of non‐drug treatments, included medications and other modalities (e.g., vitamin supplements) in their count of combinations, or did not report on combinations at all. Guidelines recommended intensity of treatment, that is, 25 hr/week for children [Maglione et al., 2012], was not achieved in our study. Even the most intense treatments have not reached this level (children aged 3–4, four or more different interventions, mean intensity 19.7 hr/week).

SLT was the most frequently used therapy in our study and was predominantly provided at public schools. That SLT was the most frequent therapy agrees with previous findings in the United States [Becerra et al., 2017] (from a 2012 survey of four Kaiser Permanente regions) and in Europe [Salomone et al., 2016]. Since communication deficits are a core symptom of ASD, the high rate of SLT is not surprising.

We classified geography based on state and county, defining six levels of urbanization from most urban/highest density to most rural/lowest density [Ingram & Franco, 2014]. The difference in frequency of “any” treatment between metropolitan and nonmetropolitan areas (96.2% vs 93.7%, OR 1.71 [95% CI 1.17, 2.45]), although significant, was less pronounced than might have been expected [Kelleher & Gardner, 2017]. This may be because the most frequently used therapies (SLT and occupational therapy) are mostly provided at public school, and are thus not dependent on infrastructure such as specialists' offices. The intensity of “any” treatment was also significantly greater in metropolitan areas than in nonmetropolitan areas; mostly driven by significant differences in the intensity of behavioral therapy, SLT, psychological, and “other” interventions. Specifically, behavioral therapy was less often used in nonmetropolitan settings, and, where used, was much less intense. Given that public school was reported as the most frequent setting for this therapy in nonmetropolitan areas, its use as a platform for care delivery probably warrants further consideration. Kelleher and Gardner [2017] have additionally suggested telehealth programs to provide better access to behavioral therapy in remote areas. The level of urbanization has an influence on the number of children diagnosed with ASD [Antezana, Scarpa, Valdespino, Albright, & Richey, 2017], also called “treated prevalence” [Mandell et al., 2016]. Hence, the true difference in unmet need between metropolitan and nonmetropolitan areas is likely underestimated in our study.

Historically, large differences have been reported for ASD‐related services between children covered under Medicaid versus those under private insurance, such that Medicaid spending was much higher [Wang et al., 2013; Zhang & Baranek, 2016]. We were able to investigate the association between frequency/intensity and type of healthcare coverage in two mutually exclusive insurance groups, namely Medicaid‐only and private employer‐based‐only. We made these groups as comparable as possible by focusing on a subgroup of children with similar characteristics and further adjusting for important confounders. No significant differences in the frequency or intensity of treatment was observed, with the exception that intensity for “other” interventions was lower in privately insured children. However, given that the sample size for this analysis was substantially smaller than for the analysis of association with geography, the results may also reflect limited statistical power, as point estimates mostly directionally favored Medicaid, except for behavioral and developmental/relationship‐based therapies.

Our study has some limitations. The use of the SPARK cohort might have introduced selection bias toward caregivers with higher motivation and higher education. This is likely to have underestimated the difference between nonmetropolitan and metropolitan areas, given the known associations between area of residence and educational level and likelihood of seeking treatment [Payakachat et al., 2018]. The possibility of recall bias cannot be ruled out as the study relied on caregiver‐reported information over the previous year. However, as most of the treatments were still ongoing, this seems to be less of a concern. Our grouping of treatment options into categories may not be universally acceptable, although a very similar grouping has been used before [Salomone et al., 2016], and caregivers may not have been able to clearly identify and distinguish the different treatment options. We also did not collect in the “Other” category further details for the response option “Other therapy not previously mentioned,” which may include those considered complementary health approaches. Finally, as this was a cross‐sectional study, it is not possible to conclude any causal relationships, particularly between geographic region and any type of healthcare coverage, although it is implied that the pattern of care is determined by geography of residence and type of coverage.

Strengths of this study lie in the recency of the data presented (2016 and 2017), and its collection from a large sample not linked to a specific provider or network of centers, and no reliance on claims data. Since most respondents reported themselves as the main care coordinator, the data are likely to be complete. The design of the survey also allowed us to capture important details including the setting of care. Finally, these data will be made available via SPARK, and will be linkable to other data collected from the same cohort.

While this study provides unprecedented detail on current non‐drug therapy in ASD for children in the United States, future research should investigate the effectiveness of those treatments in routine practice.

## Conflict of Interest

B.M., R.H., and G.L. are full‐time employees of F. Hoffmann‐La Roche Ltd., which has drugs for autism under development. B.M. holds stock options from F. Hoffmann‐La Roche Ltd. K.L. is a research consultant with the Simons Foundation Autism Research Initiative which funds and operates SPARK.

## Data Sharing

Approved researchers can obtain the SPARK population dataset described in this study by applying at https://base.sfari.org.

## Supporting information


**Appendix S1:** Supplementary Information to Caregiver‐reported non‐drug treatment patterns in children with autism: A US‐wide study nested in an online research cohort
