# Supplementary material for: Treatment patterns in children with autism in the United States
Source: Autism Res. 2019 Jan 10;12(3):517–26. doi: 10.1002/aur.2070 (PMC6519302; doi:10.1002/aur.2070)
Supplement: Supplementary file 1 — Appendix S1: Supplementary Information to Caregiver‐reported non‐drug treatment patterns in children with autism: A US‐wide study nested in an online research cohort [file AUR-12-517-s001.docx]

# Supplementary Information to Caregiver-reported non-drug treatment patterns in children with autism: A US-wide study nested in an online research cohort

Brigitta U. Monz^1^, Richard Houghton^1,2^, Kiely Law^3,4^, Georg Loss^1^

**^1^** Personalized Health Care Data Science, Real World Data, F. Hoffmann-La Roche Ltd., Grenzacherstrasse. 124, 4070 Basel, Switzerland

^2^ School CAPHRI, Department of Clinical Pharmacy & Toxicology, Maastricht UMC+, The Netherlands

**^3^** Kennedy Krieger Institute, 707 North Broadway, Baltimore, MD 21205, US

^4^ Johns Hopkins University School of Medicine, 733 North Broadway, Baltimore, MD 21205, US

## Supplementary Methods

Confounder identification using directed acyclic graphs (DAGs)

DAGs (Figure S1 and Figure S2) were used to understand the relationships among all variables (measured and unmeasured) that influence the relationship between type of healthcare coverage (“insurance” in the DAG) and treatment, or between geography and treatment. Insurance and geography were included in both DAGs. We used the backdoor test for sufficiency to determine the minimum adjustment set of variables.^1^ We graphically displayed the DAGs using free software (www.DAGitty.net).^2^ The adjustment set identified was confirmed using the graphical criteria of the backdoor test.^1^ If more than one possible set was identified, we used the set with fewer variables.

Geography as exposure was investigated using the dichotomous variable metropolitan/nonmetropolitan (designated ‘urban/rural’ in the figures), while US state was investigated as a potential confounder.

The minimum adjustment set of variables for the association between insurance and treatment (Figure S1) identified autism spectrum disorder (ASD) severity, child race, child medical comorbidity, child mental comorbidity, geography (rural/urban), household income, marital status, mother’s employment, and US state. The adjustment set in the DAG for geography (Figure S2) was empty (as no confounder was identified).

Confounder adjustment (propensity score) and modelling

*Health Care Coverage*

The minimum adjustment set of variables for the association between insurance and non-drug treatment included some variables which were systematically related to one another (e.g. insurance and household income). To balance the covariate distributions among the different insurance categories first, a propensity score methodology was employed. We restricted our analysis only to those families with an annual income between $20,000 and $99,999, because in the lower and upper strata of household income children were almost exclusively enrolled into Medicaid (<$20,000) or private provided by employer (>$99,000); meaning there was very small overlap between the populations. We excluded uninsured patients and those with missing covariates, as well as small number of responses from caregivers other than the mother. Only respondents living in states with at least 3 participants for each insurance type were included, to ensure that parameter estimates were not distorted due to small cell counts.

The primary comparison was between Medicaid-only and private insurance-only, given that we could observe sufficient overlap in the characteristics of those two groups for the minimum adjustment set of variables; and their sample size. We estimated the propensity score (probability) of receiving either of the two insurance types using logistic regression. The minimum adjustment set of variables (including interaction terms for household income, Autism Impact Measure (AIM) domain scores, and child’s other mental or medical comorbidities) was included. Then, Inverse probability weighting (IPW) methodology was applied, as we were primarily interested in the average treatment effect. Finally, we incorporated the IPWs in a series of simple logistic regression analyses which modeled the association between insurance type and presence/absence of each of the non-drug treatments.

Similarly, we incorporated the IPWs in negative binomial models that described associations between insurance type and the intensity (in hours per week) of each of the non-drug treatments. We rounded responses of ‘less than 1 hour’ and ‘don’t know’ to 1 hour and responses of ‘more than 20’ to 21 hours to have an integer outcome. The full range was therefore 0-21 hours for individual treatments and 0-126 for the ‘any’ category (the sum of all child-oriented treatments).

We repeated the analyses of the presence/absence and intensity of treatments using the ‘doubly-robust’ method^3^, i.e. including the minimum adjustment set of variables as covariates into the final models alongside the IPWs. US state was not included in the doubly-robust analyses due to small cell counts causing convergence issues with some models.

For both approaches (i.e. IPW only and double-robust), we additionally fitted models by excluding those patients with large IPW (>20), i.e. used trimming, to limit the influence of respondents with unique characteristics.^4^ We inspected IPW population characteristics (Medicaid-only vs. private insurance-only) for the variable set before and after trimming (Table S6).

*Geography*

As per the DAG (Figure S2), there was no need to adjust for any covariates when testing for associations between non-drug treatments and geography (metropolitan vs nonmetropolitan).

## Supplementary Figures

**Figure S1:** **Directed Acyclic Graph displaying the relationship between insurance (i.e. types of health care coverage) and treatment (type and intensity)**


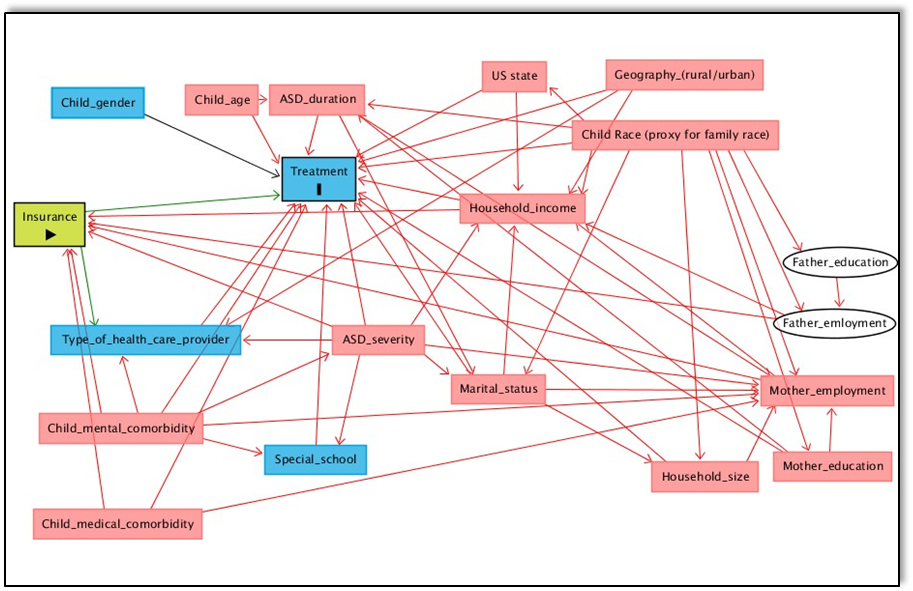


**Legend:** Blue boxes: Ancestors of outcome; red boxes: ancestor of exposure and outcome, clear boxes: unmeasured; green arrows: causal paths; red arrows: biasing paths

Note: Insurance was exposure; treatment was outcome. Other variables were investigated for confounding

**Figure S2: Directed Acyclic Graph displaying the relationship between geography (rural/urban) and treatment (type and intensity)**

*
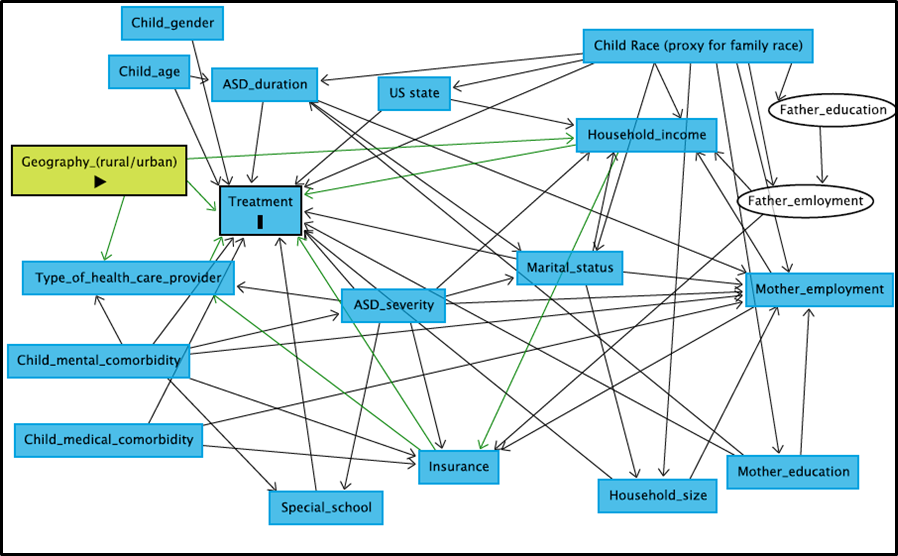
*

**Legend:** Blue boxes: Ancestors of outcome; clear boxes: unmeasured; green arrows: causal paths

Note: Geography was exposure; treatment was outcome. Other variables were investigated for confounding

**Figure S3: Participant flow**


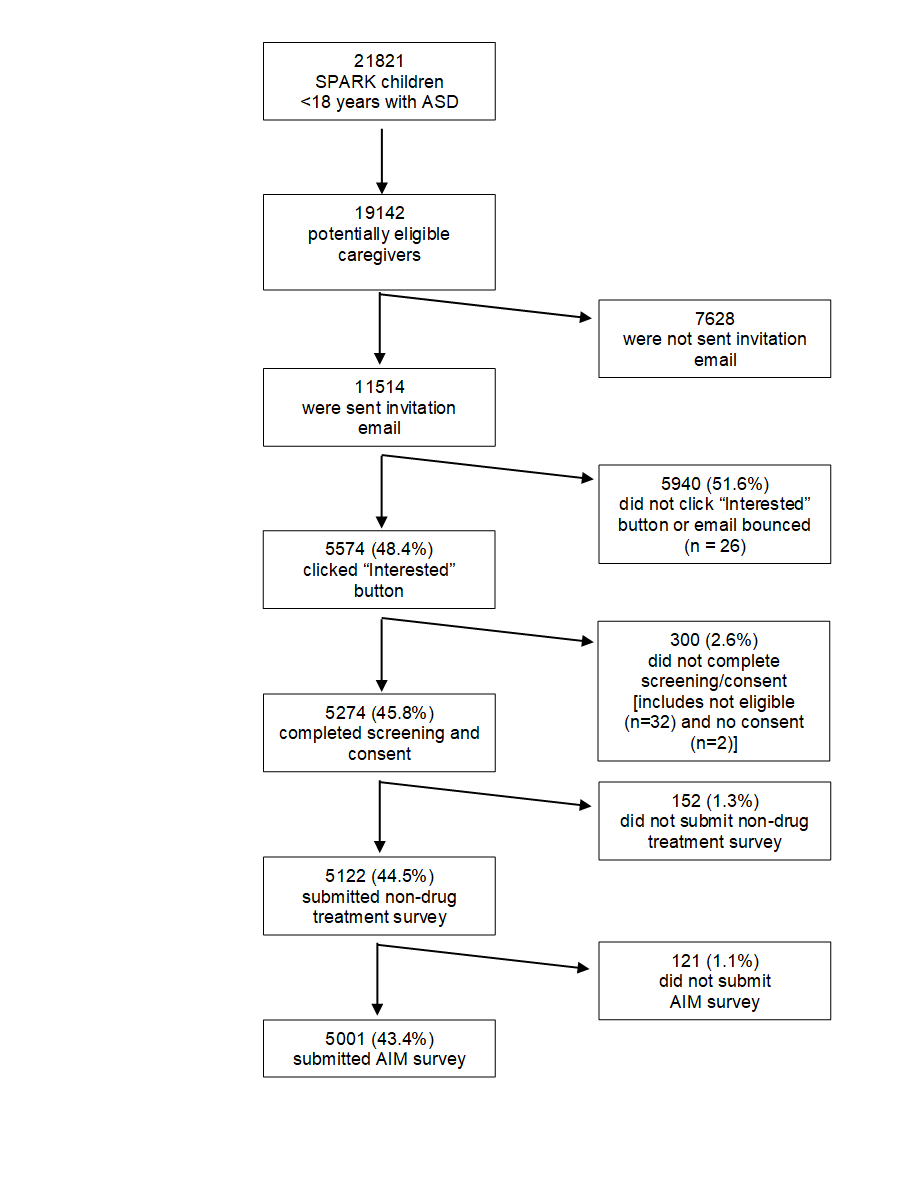


Note: Parentheses show % of total participants sent the invitation email

**Figure S4: Distribution of Hours - MSA**


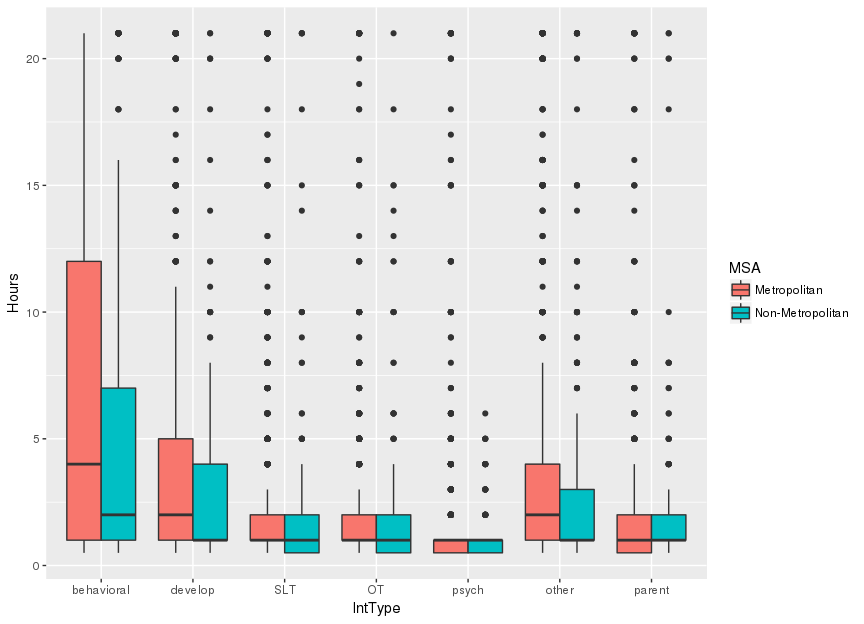


Behavioral: Behavioral intervention; Develop: Developmental and/or relationship-based intervention; IntType: Intervention Type; MSA: Metropolitan Statistical Area; OT: Occupational Therapy; Other: Other intervention; Parent: Parent/Caregiver training; Psych: Psychological intervention; SLT: Speech and language therapy

**Figure S5: Distribution of Hours: Insurance-derived**


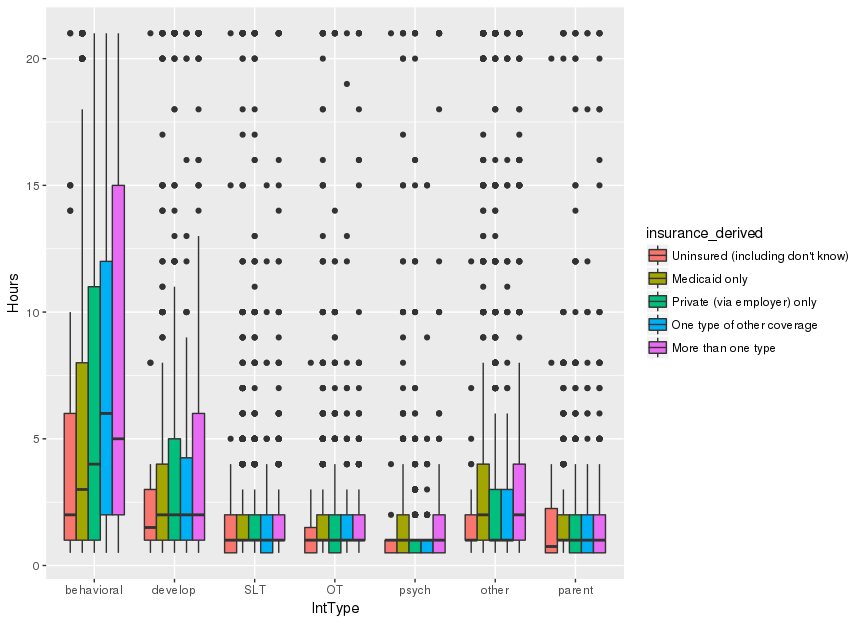


Behavioral: Behavioral intervention; Develop: Developmental and/or relationship-based intervention; IntType: Intervention Type; OT: Occupational Therapy; Other: Other intervention; Parent: Parent/ Caregiver training; Psych: Psychological intervention; SLT: Speech and language therapy

## Supplementary Tables

| **Table S1: Characteristics of children with ASD by type of school attended (post-hoc)** | | | |
| --- | --- | --- | --- |
| **Parameter** | **Category** | **n (%)^a^** | |
|  |  | **Full-time in Special-Education School** | **Not full-time in Special-Education School** |
| n | | 1109 | 3986 |
| Caregiver’s relationship to child | Parent | 1088 (98.1) | 3931 (98.6) |
|  | Legal guardian | 17 (1.5) | 37 (0.9) |
|  | Other | 4 (0.4) | 10 (0.3) |
|  | NA | 0 (0.0) | 8 (0.2) |
| Caregiver’s relationship to child, by gender | Legal guardian-Female | 17 (1.5) | 36 (0.9) |
|  | Legal guardian-Male | 0 (0.0) | 1 (0.0) |
|  | Other-Female | 4 (0.4) | 10 (0.3) |
|  | Other-Male | 0 (0.0) | 0 (0.0) |
|  | Parent-Female | 987 (89.0) | 3706 (93.0) |
|  | Parent-Male | 101 (9.1) | 213 (5.3) |
|  | Unknown | 0 (0.0) | 20 (0.5) |
| Age in years [mean (SD)] | | 8.27 (4.21) | 9.34 (3.81) |
| Age category | 3-4 years | 286 (25.8) | 396 (9.9) |
|  | 5-9 years | 404 (36.4) | 1772 (44.5) |
|  | 10-14 years | 306 (27.6) | 1325 (33.2) |
|  | 15-17 years | 112 (10.1) | 483 (12.1) |
|  | NA | 1 (0.1) | 10 (0.3) |
| Gender | Male | 903 (81.4) | 3172 (79.6) |
|  | Female | 201 (18.1) | 789 (19.8) |
|  | NA | 5 (0.5) | 25 (0.6) |
| Race/ethnicity (derived) | White/Non-Hispanic | 659 (59.4) | 2831 (71.0) |
|  | White/Hispanic | 133 (12.0) | 365 (9.2) |
|  | Non-white/Non-Hispanic | 231 (20.8) | 596 (15.0) |
|  | Non-white/Hispanic | 86 (7.8) | 194 (4.9) |
| Child’s general health status (caregiver-assessed) | Excellent | 320 (28.9) | 1370 (34.4) |
|  | Very Good | 472 (42.6) | 1694 (42.5) |
|  | Good | 266 (24.0) | 788 (19.8) |
|  | Fair | 46 (4.1) | 118 (3.0) |
|  | Poor | 5 (0.5) | 7 (0.2) |
|  | NA | 0 (0.0) | 9 (0.2) |
| Autism diagnosis | ASD | 849 (76.6) | 2920 (73.3) |
|  | Autism/Autistic Disorder | 177 (16.0) | 374 (9.4) |
|  | Asperger Syndrome | 36 (3.2) | 414 (10.4) |
|  | PDD-NOS | 43 (3.9) | 244 (6.1) |
|  | Don’t know | 4 (0.4) | 24 (0.6) |
|  | NA | 0 (0.0) | 10 (0.3) |
| Age at diagnosis | 0-2 years | 449 (40.5) | 881 (22.1) |
|  | 3-4 years | 445 (40.1) | 1543 (38.7) |
|  | 5-9 years | 174 (15.7) | 1238 (31.1) |
|  | 10-14 years | 32 (2.9) | 286 (7.2) |
|  | 15-17 years | 4 (0.4) | 23 (0.6) |
|  | 18+ years | 0 (0.0) | 0 (0.0) |
|  | NA | 5 (0.5) | 15 (0.4) |
| Time since diagnosis | 0-1 years | 248 (22.4) | 839 (21.0) |
|  | 2-3 years | 294 (26.5) | 1000 (25.1) |
|  | 4-5 years | 164 (14.8) | 714 (17.9) |
|  | 6-9 years | 215 (19.4) | 909 (22.8) |
|  | 10+ years | 184 (16.6) | 512 (12.8) |
|  | NA | 4 (0.4) | 12 (0.3) |
| Healthcare coverage (derived) | Uninsured (including don’t know) | 19 (1.7) | 67 (1.7) |
|  | Medicaid only | 366 (33.0) | 1190 (29.9) |
|  | Private (via employer) only | 420 (37.9) | 1653 (41.5) |
|  | One type of other coverage | 87 (7.8) | 330 (8.3) |
|  | More than one type | 217 (19.6) | 746 (18.7) |
| Prescription medication insurance coverage within past 12 months | Yes | 986 (88.9) | 3661 (91.8) |
|  | No | 109 (9.8) | 288 (7.2) |
|  | Don’t know | 9 (0.8) | 24 (0.6) |
|  | NA | 5 (0.5) | 13 (0.3) |
| Currently taking prescription medication | Yes | 552 (49.8) | 2114 (53.0) |
|  | No | 557 (50.2) | 1863 (46.7) |
|  | Don’t know | 0 (0.0) | 1 (0.0) |
|  | NA | 0 (0.0) | 8 (0.2) |
| Whether prescription medication being taken currently is for ASD^b^ | n | 552 | 2114 |
|  | Yes | 370 (67.0) | 1339 (63.3) |
|  | No | 174 (31.5) | 729 (34.5) |
|  | Don’t know | 6 (1.1) | 43 (2.0) |
|  | NA | 2 (0.4) | 3 (0.1) |
| Currently taking OTC medications regularly | Yes | 646 (58.3) | 2444 (61.3) |
|  | No | 463 (41.7) | 1531 (38.4) |
|  | Don’t know | 0 (0.0) | 1 (0.0) |
|  | NA | 0 (0.0) | 10 (0.3) |
| Whether OTC medications being currently taken are for ASD^c^ | n | 646 | 2444 |
|  | Yes | 216 (33.4) | 741 (30.3) |
|  | No | 421 (65.2) | 1662 (68.0) |
|  | Don’t know | 6 (0.9) | 40 (1.6) |
|  | NA | 3 (0.5) | 1 (0.0) |
| Other medical problems over the last 12 months | Yes | 515 (46.4) | 1818 (45.6) |
|  | No | 584 (52.7) | 2141 (53.7) |
|  | Don’t know | 8 (0.7) | 18 (0.5) |
|  | NA | 2 (0.2) | 9 (0.2) |
| Other mental/psychiatric problems over the last 12 months | Yes | 437 (39.4) | 1973 (49.5) |
|  | No | 636 (57.3) | 1926 (48.3) |
|  | Don’t know | 35 (3.2) | 79 (2.0) |
|  | NA | 1 (0.1) | 8 (0.2) |
| Main healthcare provider for any medical care related to ASD | Primary care physician or nurse practitioner (including general pediatrician, family doctor or internists) | 592 (53.4) | 2303 (57.8) |
|  | Developmental Pediatrician | 207 (18.7) | 643 (16.1) |
|  | Neurologist | 87 (7.8) | 207 (5.2) |
|  | Adult Psychiatrist | 8 (0.7) | 20 (0.5) |
|  | Child Psychiatrist (or dual child/adult psychiatrist) | 146 (13.2) | 585 (14.7) |
|  | ER physician (Urgent Care) | 3 (0.3) | 5 (0.1) |
|  | Other | 30 (2.7) | 83 (2.1) |
|  | No main provider | 32 (2.9) | 128 (3.2) |
|  | Don’t know | 4 (0.4) | 11 (0.3) |
|  | NA | 0 (0.0) | 1 (0.0) |
| Last appointment with main healthcare provider | Less than 3 months | 547 (49.3) | 1884 (47.3) |
|  | 3 to 6 months | 267 (24.1) | 880 (22.1) |
|  | More than 6 months, but less than 1 year | 166 (15.0) | 609 (15.3) |
|  | More than 1 year | 92 (8.3) | 467 (11.7) |
|  | NA | 37 (3.3) | 146 (3.7) |
| Whether IQ test ever given | Yes | 428 (38.6) | 1870 (46.9) |
|  | No | 535 (48.2) | 1541 (38.7) |
|  | Don’t know | 146 (13.2) | 572 (14.4) |
|  | NA | 0 (0.0) | 3 (0.1) |
| IQ test score^d^ | n | 428 | 1870 |
|  | IQ score 70 or below | 160 (37.4) | 304 (16.3) |
|  | IQ score between 71 and 99 | 92 (21.5) | 469 (25.1) |
|  | IQ score 100 or above | 76 (17.8) | 712 (38.1) |
|  | Don’t know | 99 (23.1) | 379 (20.3) |
|  | NA | 1 (0.2) | 6 (0.3) |

AIM: Autism Impact Measure; ASD: Autism Spectrum Disorder; ER: Emergency Room; IQ: Intelligence Quotient; NA: Unknown/Missing; OTC: Over the Counter; PDD-NOS: Pervasive developmental disorder-not otherwise specified; SD: Standard Deviation

1. Except where indicated otherwise
2. Only considered for those who had answered that their ASD child was currently taking prescription medication. Adjusted ‘n’ is shown.
3. Only considered for those who had answered that their ASD child was currently taking over-the-counter (non-prescription) medication. Adjusted ‘n’ is shown.
4. Only considered for those who had answered “yes” to whether the ASD child had been given an IQ test. Adjusted ‘n’ is shown.

Note: Data in this table were not used in the primary analyses

| **Table S2: Caregiver-reported types of non-drug treatments by 6-level MSA** | | | | | | | | | |
| --- | --- | --- | --- | --- | --- | --- | --- | --- | --- |
|  | **N** | **n (%)** | | | | | | | |
|  |  | **Any** | **Behavi­oral** | **D/R** | **SLT** | **OT** | **Psychological** | **Other** | **Parent/ Caregiver Training** |
| Large central metro | 1248 | 1204 (96.5) | 724 (58.0) | 330 (26.4) | 923 (74.0) | 740 (59.3) | 350 (28.0) | 841 (67.4) | 402 (32.2) |
| Large fringe metro | 1527 | 1470 (96.3) | 893 (58.5) | 392 (25.7) | 1103 (72.2) | 954 (62.5) | 447 (29.3) | 1060 (69.4) | 468 (30.6) |
| Medium metro | 938 | 898 (95.7) | 522 (55.7) | 243 (25.9) | 662 (70.6) | 540 (57.6) | 279 (29.7) | 628 (67.0) | 255 (27.2) |
| Small metro | 445 | 429 (96.4) | 239 (53.7) | 116 (26.1) | 317 (71.2) | 266 (59.8) | 134 (30.1) | 299 (67.2) | 128 (28.8) |
| Micropolitan | 369 | 345 (93.5) | 177 (48.0) | 96 (26.0) | 238 (64.5) | 212 (57.5) | 95 (25.7) | 242 (65.6) | 94 (25.5) |
| Non-core | 219 | 206 (94.1) | 96 (43.8) | 56 (25.6) | 144 (65.8) | 122 (55.7) | 62 (28.3) | 135 (61.6) | 48 (21.9) |
| Unknown | 376 | 366 (97.3) | 224 (59.6) | 115 (30.6) | 270 (71.8) | 244 (64.9) | 104 (27.7) | 266 (70.7) | 120 (31.9) |

D/R: Developmental and/or relationship-based intervention; MSA: Metropolitan Statistical Area; OT: Occupational therapy; SLT: Speech and Language Therapy

| **Table S3: Associations between different types of non-drug therapy in the last 12 months** | | | | | | | | |
| --- | --- | --- | --- | --- | --- | --- | --- | --- |
| **Variable** | **OR (95% CI) for Association Between Interventions** | | | | | | | |
|  | **Behavioral** | **D/R** | **SLT** | **OT** | **Psycho-logical** | **Other** | **Parent/ Caregiver Training** | **Special School Only** |
| Behavioral | NA | NA | NA | NA | NA | NA | NA | NA |
| D/R | 2.05 (1.80,2.35) | NA | NA | NA | NA | NA | NA | NA |
| SLT | 1.59 (1.41,1.80) | 1.31  (1.14,1.51) | NA | NA | NA | NA | NA | NA |
| OT | 2.04 (1.82,2.28) | 1.44  (1.26,1.64) | 6.78 (5.93,7.76) | NA | NA | NA | NA | NA |
| Psychological | 1.59 (1.40,1.80) | 1.79  (1.57,2.04) | 0.41 (0.36,0.46) | 0.72 (0.64,0.82) | NA | NA | NA | NA |
| Other | 1.69 (1.51,1.91) | 2.40  (2.07,2.79) | 1.26 (1.11,1.43) | 1.60 (1.42,1.80) | 2.12 (1.84,2.44) | NA | NA | NA |
| Parent/caregiver training | 3.06 (2.68,3.50) | 1.81  (1.59,2.06) | 1.49 (1.30,1.71) | 1.66 (1.47,1.89) | 1.72 (1.51,1.96) | 1.89 (1.65,2.17) | NA | NA |
| Special-education school only | 1.97 (1.72,2.28) | 1.34  (1.16,1.55) | 3.06 (2.56,3.69) | 2.17 (1.87,2.52) | 0.78 (0.67,0.90) | 1.11 (0.96,1.28) | 1.28 (1.11,1.48) | NA |
|  |  |  |  |  |  |  |  |  |

CI: Confidence interval; D/R: Developmental and/or relationship-based intervention; NA: Not applicable; OR: Odd’s Ratio; OT: Occupational Therapy; SLT: Speech and Language Therapy

| **Table S4: Patterns of treatments: non-drug therapy for all groups with >2%** | | | | | | | | |
| --- | --- | --- | --- | --- | --- | --- | --- | --- |
| **Behavioral** | **D/R** | **SLT** | **OT** | **Psychological** | **Other** | **Parent/ Caregiver Training** | **n** | **%** |
| **Overall (n =5,122)** |  |  |  |  |  |  |  |  |
| Y | N | Y | Y | N | Y | N | 362 | 7.1 |
| N | N | Y | Y | N | Y | N | 302 | 5.9 |
| N | N | Y | Y | N | N | N | 243 | 4.7 |
| Y | N | Y | Y | N | Y | Y | 235 | 4.6 |
| N | N | N | N | N | N | N | 185 | 3.6 |
| Y | N | Y | Y | N | N | N | 180 | 3.5 |
| N | N | Y | N | N | Y | N | 153 | 3.0 |
| Y | Y | Y | Y | N | Y | N | 150 | 2.9 |
| N | N | Y | N | N | N | N | 146 | 2.9 |
| Y | Y | Y | Y | N | Y | Y | 143 | 2.8 |
| N | N | N | N | N | Y | N | 140 | 2.7 |
| **By geography: Metropolitan** **(n=588)** | | | | | | | | |
| Y | N | Y | Y | N | Y | N | 305 | 7.3 |
| N | N | Y | Y | N | Y | N | 225 | 5.4 |
| Y | N | Y | Y | N | Y | Y | 202 | 4.9 |
| N | N | Y | Y | N | N | N | 186 | 4.5 |
| Y | N | Y | Y | N | N | N | 152 | 3.7 |
| N | N | N | N | N | N | N | 142 | 3.4 |
| N | N | Y | N | N | Y | N | 126 | 3.0 |
| Y | Y | Y | Y | N | Y | N | 124 | 3.0 |
| Y | Y | Y | Y | N | Y | Y | 121 | 2.9 |
| N | N | Y | N | N | N | N | 112 | 2.7 |
| N | N | N | N | N | Y | N | 111 | 2.7 |
| Y | N | Y | Y | Y | Y | Y | 87 | 2.1 |
| Y | N | Y | Y | Y | Y | N | 86 | 2.1 |
| **By geography: Nonmetropolitan (n=4,158)** | | | | | | | | |
| N | N | Y | Y | N | Y | N | 47 | 8.0 |
| N | N | Y | Y | N | N | N | 41 | 7.0 |
| N | N | N | N | N | N | N | 35 | 6.0 |
| Y | N | Y | Y | N | Y | N | 31 | 5.3 |
| N | N | Y | N | N | N | N | 28 | 4.8 |
| N | N | N | N | N | Y | N | 23 | 3.9 |
| N | N | Y | N | N | Y | N | 19 | 3.2 |
| Y | N | Y | Y | N | N | N | 16 | 2.7 |
| Y | N | Y | N | N | Y | N | 14 | 2.4 |
| Y | N | Y | Y | Y | Y | N | 13 | 2.2 |

D/R: Developmental and/or relationship-based intervention; N: No; OT: Occupational Therapy; SLT: Speech and Language Therapy; Y: Yes

| **Table S5: Median Intensity and setting of non-drug treatments by 6-level MSA** | | | | | | | | | | | | | | | | | | | | |
| --- | --- | --- | --- | --- | --- | --- | --- | --- | --- | --- | --- | --- | --- | --- | --- | --- | --- | --- | --- | --- |
|  | **N (any)** | **Any** | **Behavioral** | | | **D/R** | | | **SLT** | | | **OT** | | | **Psychological** | | | **Other** | | |
|  |  | **Int** | **Int** | **IGR** | **SNR** | **Int** | **IGR** | **SNR** | **Int** | **IGR** | **SNR** | **Int** | **IGR** | **SNR** | **Int** | **IGR** | **SNR** | **Int** | **IGR** | **SNR** |
| Large central metro | 1204 | 7 | 6 | 1.99 | 0.63 | 2 | 1.30 | 0.93 | 1 | 1.67 | 1.61 | 1 | 2.72 | 1.16 | 1 | 2.66 | 0.43 | 2 | 1.08 | 0.92 |
| Large fringe metro | 1470 | 6.5 | 4 | 2.04 | 0.66 | 2 | 1.29 | 1.04 | 1 | 1.50 | 1.89 | 1 | 2.64 | 1.40 | 1 | 3.01 | 0.49 | 2 | 1.14 | 1.03 |
| Medium metro | 898 | 6 | 3 | 2.18 | 0.64 | 2 | 1.29 | 1.02 | 1 | 1.82 | 1.87 | 1 | 3.04 | 1.27 | 1 | 3.78 | 0.33 | 2 | 1.21 | 0.97 |
| Small metro | 429 | 6 | 3 | 2.42 | 0.70 | 2 | 1.41 | 1.04 | 1 | 2.08 | 1.88 | 1 | 3.46 | 1.57 | 1 | 3.69 | 0.31 | 2 | 1.32 | 1.12 |
| Micropolitan | 345 | 4.5 | 2 | 3.00 | 0.57 | 2 | 1.44 | 1.02 | 1 | 2.14 | 1.94 | 1 | 3.06 | 1.60 | 1 | 5.81 | 0.32 | 1 | 1.52 | 0.97 |
| Non-core | 206 | 4 | 2 | 3.68 | 0.53 | 1 | 2.24 | 0.92 | 1 | 3.22 | 1.82 | 1 | 5.85 | 1.58 | 1 | 3.62 | 0.44 | 1 | 1.75 | 1.15 |
| Unknown | 366 | 5.5 | 3 | 2.04 | 0.66 | 2 | 1.48 | 0.90 | 1 | 1.81 | 1.61 | 1 | 3.28 | 1.29 | 1 | 3.72 | 0.39 | 1 | 1.28 | 0.98 |

D/R: Developmental and/or relationship-based intervention; IGR: individual to group sessions ratio, Int: intensity (median hours/week); MSA: Metropolitan Statistical Area; OT: Occupational therapy; SLT: Speech and Language Therapy, SNR: school to not in school sessions ratio

| **Table S6: Association of caregiver-reported type and intensity of non-drug therapy with insurance (trimmed)** | | |
| --- | --- | --- |
| **Non-drug Therapy Type** | **Insurance***  **(Only private via employer vs. only Medicaid)**  **N=1,625**** | |
|  | **PS-adjusted** | **Double-adjusted** |
| **Association with type of therapy OR (95% CI)** | | |
| Any | 0.57 (0.31,1.04) | 0.53 (0.28,0.99) |
| Behavioral | 0.84 (0.64,1.10) | 0.84 (0.63,1.13) |
| Developmental/ relationship | 1.27 (0.94,1.72) | 1.32 (0.95,1.83) |
| SLT | 0.91 (0.68,1.21) | 0.90 (0.64,1.28) |
| Occupational | 0.86 (0.66,1.14) | 0.85 (0.62,1.16) |
| Psychological | 0.80 (0.61,1.07) | 0.77 (0.55,1.06) |
| Other | 0.89 (0.67,1.18) | 0.85 (0.62,1.17) |
| **Association with intensity of therapy RR (95% CI)** | | |
| Any | 0.94 (0.80,1.11) | 0.89 (0.77,1.03) |
| Behavioral | 1.04 (0.84,1.28) | 0.98 (0.78,1.22) |
| Developmental/ relationship | 1.49 (1.03,2.17) | 1.21 (0.86,1.71) |
| SLT | 0.94 (0.74,1.21) | 0.90 (0.76,1.08) |
| Occupational | 0.86 (0.68,1.08) | 0.89 (0.72,1.10) |
| Psychological | 0.95 (0.66,1.38) | 0.92 (0.63,1.33) |
| Other | 0.71 (0.58,0.86) | 0.69 (0.56,0.85) |

CI: Confidence interval; OR: Odds Ratio; PS: propensity score; RR: Rate ratio; SLT: Speech and language therapy

*Models were adjusted for below variables using propensity score inverse probability weighting (adjusted and double adjusted: see methods section and Supplementary Appendix)

Variables for adjustment: AIM domain scores (continuous), child race/ethnicity (White/Hispanic, White/Non-Hispanic, Non-White/Hispanic, Non-White/Non-Hispanic), child other medical problems (yes/no), child other mental health or psychiatric problems (yes/no), geography (nonmetropolitan/ metropolitan), household income (four strata, ≥$20,000 to ≤$99,999), marital status (married/living with partner yes/no), mother employment (work full time/part time yes/no; excluded fathers), US state (excluded states with n<3 for Medicaid and private employer insurance).

** 7 participants trimmed (2 from only Medicaid group, 5 from only private via employer group)

| **Table S7 : Association of caregiver-reported type and intensity of non-drug therapy with geography (6-level MSA)** | | | | | | | |
| --- | --- | --- | --- | --- | --- | --- | --- |
| **Geography**  **(non-core=reference)** | **Any** | **Behavioral** | **Developmental/**  **relationship** | **SLT** | **Occupational** | **Psychological** | **Other** |
| **Association with type of therapy: OR (95% CI)** | | | | | | | |
| Large central metro | 1.73 (0.88,3.17) | 1.77 (1.33,2.37) | 1.05 (0.76,1.46) | 1.48 (1.08,2.00) | 1.16 (0.87,1.55) | 0.99 (0.72,1.37) | 1.29 (0.95,1.73) |
| Large fringe metro | 1.63 (0.84,2.93) | 1.80 (1.36,2.41) | 1.01 (0.73,1.40) | 1.35 (1.00,1.82) | 1.32 (0.99,1.76) | 1.05 (0.77,1.44) | 1.41 (1.05,1.89) |
| Medium metro | 1.42 (0.72,2.63) | 1.61 (1.20,2.17) | 1.02 (0.73,1.43) | 1.25 (0.91,1.70) | 1.08 (0.80,1.45) | 1.07 (0.78,1.49) | 1.26 (0.93,1.71) |
| Small metro | 1.69 (0.79,3.58) | 1.49 (1.07,2.06) | 1.03 (0.71,1.49) | 1.29 (0.91,1.82) | 1.18 (0.85,1.64) | 1.09 (0.77,1.57) | 1.27 (0.91,1.78) |
| Micropolitan | 0.91 (0.44,1.79) | 1.18 (0.84,1.65) | 1.02 (0.70,1.51) | 0.95 (0.66,1.34) | 1.07 (0.77,1.50) | 0.88 (0.60,1.28) | 1.19 (0.84,1.68) |
| **Association with intensity of therapy: RR (95% CI)** | | | | | | | |
| Large central metro | 1.67 (1.43,1.94) | 2.43 (1.83,3.17) | 1.48 (0.95,2.23) | 1.37 (1.15,1.65) | 1.29 (1.05,1.58) | 1.65 (1.17,2.32) | 1.24 (1.00,1.53) |
| Large fringe metro | 1.50 (1.29,1.74) | 1.95 (1.48,2.54) | 1.34 (0.87,2.01) | 1.36 (1.14,1.64) | 1.30 (1.06,1.60) | 1.43 (1.02,2.01) | 1.24 (1.00,1.52) |
| Medium metro | 1.44 (1.23,1.67) | 1.86 (1.40,2.45) | 1.22 (0.78,1.86) | 1.27 (1.05,1.53) | 1.30 (1.05,1.60) | 1.48 (1.04,2.09) | 1.21 (0.97,1.50) |
| Small metro | 1.55 (1.31,1.84) | 1.73 (1.27,2.34) | 1.71 (1.06,2.73) | 1.49 (1.22,1.83) | 1.45 (1.15,1.81) | 1.67 (1.14,2.44) | 1.32 (1.04,1.67) |
| Micropolitan | 1.23 (1.03,1.46) | 1.31 (0.95,1.80) | 1.22 (0.74,1.98) | 1.25 (1.01,1.54) | 1.41 (1.12,1.78) | 1.08 (0.72,1.61) | 1.07 (0.84,1.38) |

CI: Confidence interval; OR: Odds Ratio; RR: Rate ratio; SLT: Speech and language therapy

| **Table S8: Comparison of Weighted Populations with Medicaid vs. Private Insurance Provided by Employer, Following IPW (with and Without Trimming)** | | | | | | | | | |
| --- | --- | --- | --- | --- | --- | --- | --- | --- | --- |
| **Variable** |  | **Untrimmed** | | | | **Trimmed** | | | |
|  | **Variable Level** | **Medicaid** | **Private provided by employer** | **p-value** | **Standardized Mean Difference** | **Medicaid** | **Private provided by employer** | **p-value** | **Standardized Mean Difference** |
| n |  | 1700.27 | 1615.58 |  |  | 1620.23 | 1403.06 |  |  |
| AIM domain scores, mean (SD) | | | | | | | | | |
| Repetitive Behavior | NA | 40.98 (14.83) | 41.16 (13.31) | 0.902 | 0.013 | 42.08 (14.29) | 42.04 (13.41) | 0.967 | 0.003 |
| Communication | NA | 29.75 (11.56) | 29.71 (11.13) | 0.961 | 0.004 | 30.06 (11.75) | 29.88 (11.64) | 0.818 | 0.015 |
| Atypical Behavior | NA | 34.42 (10.31) | 34.79 (9.77) | 0.720 | 0.036 | 35.16 (9.99) | 34.49 (9.72) | 0.315 | 0.069 |
| Social Reciprocity | NA | 26.97 (7.23) | 26.94 (7.28) | 0.952 | 0.005 | 26.87 (7.38) | 26.58 (7.31) | 0.565 | 0.039 |
| Peer Interaction | NA | 22.47 (7.15) | 22.85 (6.92) | 0.577 | 0.054 | 22.77 (7.19) | 22.68 (6.86) | 0.849 | 0.013 |
| Child other medical problems (%) | No | 911.9 (53.6) | 820.9 (50.8) | 0.561 | 0.057 | 911.9 (56.3) | 800.1 (57.0) | 0.828 | 0.015 |
|  | Yes | 788.3 (46.4) | 794.7 (49.2) |  |  | 708.3 (43.7) | 603.0 (43.0) |  |  |
| Child other mental health or psychiatric problems (%) | No | 889.9 (52.3) | 829.7 (51.4) | 0.836 | 0.020 | 831.7 (51.3) | 723.5 (51.6) | 0.945 | 0.005 |
|  | Yes | 810.3 (47.7) | 785.9 (48.6) |  |  | 788.5 (48.7) | 679.5 (48.4) |  |  |
| Child race/ethnicity (%) | White/Non-Hispanic | 1115.5 (65.6) | 1029.1 (63.7) | 0.884 | 0.078 | 1057.3 (65.3) | 964.4 (68.7) | 0.771 | 0.080 |
|  | White/ Hispanic | 199.7 (11.7) | 208.4 (12.9) |  |  | 199.7 (12.3) | 146.1 (10.4) |  |  |
|  | Non-white/Non-Hispanic | 281.2 (16.5) | 297.9 (18.4) |  |  | 259.3 (16.0) | 212.4 (15.1) |  |  |
|  | Non-white/ Hispanic | 103.9 (6.1) | 80.2 (5.0) |  |  | 103.9 (6.4) | 80.2 (5.7) |  |  |
| MSA (%) | Metropolitan | 1447.4 (85.1) | 1328.3 (82.2) | 0.502 | 0.079 | 1367.4 (84.4) | 1222.0 (87.1) | 0.236 | 0.077 |
|  | Nonmetropolitan | 252.9 (14.9) | 287.2 (17.8) |  |  | 252.9 (15.6) | 181.0 (12.9) |  |  |
| Household income (%) | $20,000-34,999 | 433.3 (25.5) | 437.7 (27.1) | 0.893 | 0.059 | 433.3 (26.7) | 225.2 (16.1) | 0.011 | 0.265 |
|  | $35,000-49,999 | 321.2 (18.9) | 304.4 (18.8) |  |  | 321.2 (19.8) | 304.4 (21.7) |  |  |
|  | $50,000-74,999 | 496.3 (29.2) | 485.1 (30.0) |  |  | 496.3 (30.6) | 485.1 (34.6) |  |  |
|  | $75,000-99,999 | 449.5 (26.4) | 388.3 (24.0) |  |  | 369.5 (22.8) | 388.3 (27.7) |  |  |
| Married or living with partner (%) | Yes | 1296.2 (76.2) | 1296.1 (80.2) | 0.323 | 0.097 | 1274.4 (78.7) | 1104.4 (78.7) | 0.984 | 0.001 |
|  | No | 404.0 (23.8) | 319.4 (19.8) |  |  | 345.8 (21.3) | 298.6 (21.3) |  |  |
| Mother’s employment (work full time/part time) (%) | Yes | 1019.9 (60.0) | 948.2 (58.7) | 0.782 | 0.026 | 939.8 (58.0) | 885.9 (63.1) | 0.128 | 0.105 |
|  | No | 680.4 (40.0) | 667.4 (41.3) |  |  | 680.4 (42.0) | 517.2 (36.9) |  |  |

IPW: Inverse probability weighting; MSA: Metropolitan statistical area; N/A: Not applicable.

Data on US state not shown, but included in the propensity score calculation

## Supplementary References

1. Greenland S, Pearl J, Robins JM. Causal diagrams for epidemiologic research. Epidemiology 1999;10:37-48
2. Textor J, Hardt J, Knüppel S. DAGitty: a graphical tool for analyzing causal diagrams. Epidemiology 2011;22:745
3. Funk, M.J., Westreich, D, Wiesen, et.al. Doubly Robust Estimation of Causal Effects. Am J Epidemiol 2011;173:761–767
4. Lee BK, Lessler J, Stuart EA. Weight trimming and propensity score weighting. PloS One 2011;6:e18174

## Questionnaire

| Study Title (Public) | | Understanding Non-drug Treatment and Care for Children with ASD | | |
| --- | --- | --- | --- | --- |
| Survey Title  (Survey 1 of 2) | | ASD Therapies and Treatments Questionnaire | | |
| Description and Instructions | | Thank you for participating in this study. The information you provide about yourself, your family and your child with Autism Spectrum Disorder (ASD) will help researchers better understand how families across the United States access and use ASD therapies and treatments.  This study includes two surveys. The **first survey** will ask about therapies and treatments that your child has received during the past 12 months to help with ASD symptoms and behaviors. The **second survey**, called the Autism Impact Measure (AIM), focuses on the frequency and severity of your child’s ASD symptoms over the past two weeks. It will take approximately 20 minutes to complete both surveys. Upon completion of the study, you will receive a $20 Amazon gift code via email.  Only one child per family is eligible to participate in the study. If you have 2 or more children with ASD registered in SPARK, the name of the eligible child has been automatically inserted into the survey questions below.  *(Note: Your child’s name, as registered in SPARK, has automatically been inserted into the questions below. If the name is incorrect or the child does NOT have autism spectrum disorder, please contact* ***REDACTED****)* | | |
| SECTION TITLE | | **Section 1: Caregiver and Household Information** | | |
| DESCRIPTION/INSTRUCTIONS | | The following questions are about you and your household. | | |
| Item/Q # | Variable name | Question Text | Question Type and Response Options | Skip/display rules |
| 1 | DOB_parent | In what year were you born? | [Drop down]  from 1999 - 1917 |  |
| 2 | gender_parent | What is your gender? | [single response]  1=Female  2=Male |  |
| 3 | marital_status | Which of the following best describes you? | [single response]  1=Married  2=Single and never married  3=Living with partner  4=Divorced  5=Separated  6=Widowed  999=Prefer not to answer |  |

| Item/Q # | Variable name | Question Text | Question Type and Response Options | Skip/display rules |
| --- | --- | --- | --- | --- |

| 4 | education | What was the highest grade of school you completed? | [single response]  1= 8^th^ grade or less  2 = Some high school  3 = High school degree or equivalent  4= Trade or vocational school  4 = Some college  5 = Associate’s degree  6 = Bachelor’s degree  7 = Graduate or professional degree  999 = Prefer not to answer |  |
| --- | --- | --- | --- | --- |
| 5 | employment | Which of the following best describes your current employment? | [single response]  1=Work full-time (35 hours or more per week)  2=Work part-time (less than 35 hours per week)  3=Full-time homemaker  4=Student  5=Retired  6=Unemployed  777=Other  999=Prefer not to answer |  |
| 6 | race_parent | Which of the following describes your race? *Please check all that apply.* | [multiselect checkbox ]  1=Black or African-American  2=White or Caucasian  3=Asian or Pacific Islander  4=Native American  777=Other  999=Prefer not to answer |  |
| 7 | ethnicity_parent | Are you of Hispanic or Latino origin? | [single response]  1=Yes  0=No  999=Prefer not to answer |  |
| 8 | children_num | How many children (under 18) live in your household? | [single response]  1=1  2=2  3=3  4=4  5=5 or more |  |

| Item/Q # | Variable name | Question Text | Question Type and Response Options | Skip/display rules |
| --- | --- | --- | --- | --- |

| 9 | household_num | | How many people live in your household (including yourself and your child with ASD)? | | [single response]  2=2  3=3  4=4  5=5  6=6 or more | |  | |
| --- | --- | --- | --- | --- | --- | --- | --- | --- |
| 10 | ASD_num | | How many children (under 18) with ASD live in your household? | | [single response]  1=1  2=2  3=3  4=4  5=5 or more | |  | |
| 11 | address_state | | Where do you live? Select State. | | State: [Drop down] | |  | |
| 11.1 | address_county | | Select County (or city if independent): | | County: [Drop down] | |  | |
| 12 | health_parent | | In general, would you say your health is? | | [single response]  1=Excellent  2=Very good  3=Good  4=Fair  5=Poor | |  | |
| 13 | income | | What was your total household income in 2016? | | [single response]  1=Less than $20,000  2=$20,000 - $34,999  3=$35,000 - $49,999  4=$50,000 – $74,999  5=$75,000 - $99,999  6=$100,000 - $124,999  7=$125,000 – $149,999  8=$150,000 or more  999=Prefer not to answer | |  | |
| SECTION TITLE | | | **Section 2: Child with ASD Information** | | | | | |
| DESCRIPTION/INSTRUCTIONS | | | The following questions are about your child with ASD. | | | | | |
| 14 | | relationship | What is your relationship with [child FN]? | | [single response]  1=Parent  2=Legal guardian  777=Other | |  | |
| Item/Q # | | Variable name | | Question Text | | Question Type and Response Options | | Skip/display rules |
| 15 | | DOB_child | | In what year was [child FN] born? | | [Drop down]  from 2014 to 1999 | |  |
| 16 | | gender_child | | What is [child FN]’s gender? | | [single response]  1=Female  2=Male | |  |
| 17 | | race_child | | Which of the following describes [child FN]’s race?  *Please check all that apply.* | | [multiselect checkbox]  1=Black or African-American  2=White or Caucasian  3=Asian or Pacific Islander  4=Native American  777=Other  999=Prefer not to answer | |  |
| 18 | | ethnicity_child | | Is [child FN] of Hispanic or Latino origin? | | [single response]  1=Yes  0=No  999=Prefer not to answer | |  |
| 19 | | health_child | | In general, would you say [child FN]’s health is? | | [single response]  1=Excellent  2=Very good  3=Good  4=Fair  5=Poor | |  |
| 20 | | ASD_diagnosis | | What is [child FN]’s current ASD diagnosis? | | [single response]  1=Autism Spectrum Disorder (ASD)  2=Autism/Autistic Disorder  3=Asperger Syndrome  4=Pervasive Developmental Disorder-Not Otherwise Specified (PDD-NOS)  888=Don’t know | |  |
| 21 | | year_diagnosis | | When was [child FN] first diagnosed with ASD? | | [Drop down]  from 1999 to 2017 | |  |
| Item/Q # | | Variable name | | Question Text | | Question Type and Response Options | | Skip/display rules |
| 22 | | insurance | | What type of health care coverage/insurance has [child FN] had in the past 12 months? *Select all that apply.* | | [multiselect checkbox]  1=Uninsured  2=Private insurance provided by parent/guardian’s employer  3=Private insurance purchased in the open market  4=Private insurance purchased from a state-based health exchange (as part of the Affordable Care Act)  5=Medicaid  6=Military health care  777=Other  888=Don’t know | |  |
| 23 | | drug_coverage | | Did [child FN] have prescription medication coverage (even if not used) during the past 12 months? | | [single response]  1=Yes  0=No  888=Don’t know | |  |
| 24 | | drug_rx | | Is [child FN] currently taking any prescription medications? | | [single response]  1=Yes  0=No  888=Don’t know | |  |
| 24.1 | | ASD_drug_rx | | Is [child FN] currently taking any prescription medications for ASD symptoms and behaviors? | | [single response]  1=Yes  0=No  888=Don’t know | | Display if Q24=1 |
| 25 | | drug_otc | | Is [child FN] currently taking any over-the-counter (non-prescription) medications on a regular basis? *Examples include over-the-counter allergy medicines, stool softeners, vitamins and supplements, pain relievers, melatonin, etc.* | | [single response]  1=Yes  0=No  888=Don’t know | |  |
| 25.1 | | ASD_drug_otc | | Is [child FN] currently taking any over-the-counter (non-prescription) medications on a regular basis for ASD symptoms and behaviors**?** | | [single response]  1=Yes  0=No  888=Don’t know | | Display if Q25=1 |
| Item/Q # | | Variable name | | Question Text | | Question Type and Response Options | | Skip/display rules |
| 26 | | other_mental | | Other than his/her ASD, does [child FN] have any other **mental health or psychiatric problems** that have required treatment and/or monitoring during the past 12 months? *Examples include anxiety disorder, attention deficit hyperactivity disorder (ADHD), mood disorders, eating disorders, etc.* | | [single response]  1=Yes  0=No  888=Don’t know | |  |
| 27 | | other_medical | | Other than his/her ASD, does [child FN] have any other **medical problems** that have required treatment and/or monitoring during the past 12 months? *Examples include seizures, asthma, allergies, chronic constipation, sleep disorders, anemia, etc.* | | [single response]  1=Yes  0=No  888=Don’t know | |  |
| 28 | | medical_who | | Who is the main health care provider for any medical care related to [Child FN]’s ASD? | | [single response]  1=Primary care physician or nurse practitioner (including general pediatrician, family doctor or internists)  2=Developmental Pediatrician  3=Neurologist  4=Adult Psychiatrist  5=Child Psychiatrist (or dual child/adult psychiatrist)  6=ER physician (Urgent Care)  777=Other  7= No main provider  888=Don’t know | |  |
| 28.1 | | last_appointment | | When was the last time you had an appointment with this health care provider for medical care related to [Child FN]’s ASD? | | [single response]  1=Less than 3 months  2=3 to 6 months  3=More than 6 months, but less than 1 year  4=More than 1 year | | Display if Q28 ≠ 7 or 888 |
| 29 | | IQ_test | | Has [child FN] ever been given an intelligence (IQ) test? | | [single response]  1=Yes  0=No  888=Don’t know | |  |
| Item/Q # | | Variable name | | Question Text | | Question Type and Response Options | | Skip/display rules |
| 29.1 | | IQ_score | | What was the result of [child FN}’s most recent intelligence (IQ) test? | | [single response]  1=IQ score 70 or below  2=IQ score between 71 and 99  3=IQ score 100 or above  888=Don’t know | | Display if Q29=1 |
| 30 | | school | | Does [Child FN] attend a school with special education students ONLY? | | [single response]  1=Yes  0=No  888=Don’t know | |  |
| 30.1 | | classroom | | In the past 12 months, during an average school day, how much time does [child FN] spend in a classroom with typically developing peers? | | [single response]  1=More than 60%  2=More than 30%, less than 60%  3=less than 30%  888=Don’t know | | Display if Q31=0 |
| SECTION TITLE | | | | **Section 3: Treatments and Therapies for ASD** | | | | |
| DESCRIPTION/INSTRUCTIONS | | | | The following questions are about the treatments and therapies your child with ASD has received **in the past 12 months**. We have included descriptions and examples, as needed, to help explain each type of treatment and/or therapy.  For each question, only include information about treatments and therapies that were **conducted or delivered by a trained therapist or other professional**. Do not count hours that you, as a parent or caregiver, may have implemented the therapy at home.  We understand that it may be difficult to remember all the details. Please answer the questions as best as you can. | | | | |
| Section 3: Page 1 | | | | **Behavioral therapy** encourages appropriate behaviors (such as getting dressed or talking to other people) and discourages inappropriate behaviors (such as self-harm or aggression towards others). Desired behaviors are often broken down into small tasks which are then taught in a very structured way.  *Examples include Applied Behavior Analysis (ABA), Discrete Trial Training/Teaching (DTT), Pivotal Response Treatment (PVT), Verbal Behavior (VB) Therapy/Approach* | | | | |
| 31 | | behavioral_yn | | In the past 12 months, has [child FN] received any behavioral therapy? | | [single response]  1=Yes  0=No  888=Don’t know | |  |
| Item/Q # | | Variable name | | Question Text | | Question Type and Response Options | | Skip/display rules |
| 31.1 | | behavioral_location | | In the past 12 months, where did [child FN] receive behavioral therapy?  *Check all that apply or “Don’t know.”* | | [multiselect checkbox]  1=Home  2=Public school or pre-school program  3=Private school or pre-school program  4=Outpatient medical/autism clinic  5=Private therapist/clinician office  6=Daycare or before/after-school program  7=Camp  777=Other  888=Don’t know | | Display if Q31=1 |
| 31.2 | | behavioral_months | | During this time period, how many **months** did [child FN] receive behavioral therapy?  *Round to the nearest full month.* | | [Drop down]  1, 2, 3, 4, 5, 6, 7, 8, 9, 10, 11, 12  888=Don’t know | | Display if Q31=1 |
| 31.3 | | behavioral_hours | | For each active month, how many **hours per week**, on average, did [child FN] receive behavioral therapy?  *For example, 1 hour of therapy every 2 weeks, would mean 0.5 hours per week.* | | [Drop down]  0=less than 1 hour  1, 2, 3, 4, 5, 6,….20  21=more than 20  888=Don’t know | | Display if Q31=1 |
| 31.4 | | behavioral_sessions | | During this time period, how did [child FN] receive behavioral therapy?  *Check all that apply or “Don’t know.”* | | [multiselect checkboxes]  1=Individual sessions  2=Group sessions  777=Other  888=Don’t know | | Display if Q31=1 |
| 31.5 | | behavioral_current | | Is [child FN] still receiving behavioral therapy? | | [single response]  1=Yes  0=No  888=Don’t know | | Display if Q31=1 |
| Item/Q # | | Variable name | | Question Text | | Question Type and Response Options | | Skip/display rules |
| 31.6 | | behavioral_end | | Which of the following best describes why the behavioral therapy ended? | | [single response]  1=Scheduled to end after a certain time period or number of sessions  2=Not satisfied with progress/outcomes  3=Not satisfied with program/provider  4=Program/provider no longer available  5=Too expensive  6=Too burdensome  7=Child refused to go or participate  8=Child no longer needed  9=Switched to another option  777=Other  888=Don’t know | | Display if Q31.5=0 |
| Section 3: Page 2 | | | | **Developmental and/or relationship-based interventions** are designed to target the core challenges within each individual with autism rather than his/her outward behaviors.  *Examples include Developmental, Individual-differences, Relationship-based model (DIR/Floortime), Relationship Development Intervention (RDI), Gentle Teaching, Responsive Teaching, Son-Rise Program, Early Start Denver Model (ESDM)* | | | | |
| 32 | | develop_yn | | In the past 12 months, has [child FN] received any Developmental and/or relationship-based interventions? | | [single response]  1=Yes  0=No  888=Don’t know | |  |
| 32.1 | | develop_location | | In the past 12 months, where did [child FN] receive developmental and/or relationship-based interventions?  *Check all that apply or “Don’t know.”* | | [multiselect checkbox]  1=Home  2=Public school or pre-school program  3=Private school or pre-school program  4=Outpatient medical/autism clinic  5=Private therapist/clinician office  6=Daycare or before/after-school program  7=Camp  777=Other  888=Don’t know | | Display if Q32=1 |
| Item/Q # | | Variable name | | Question Text | | Question Type and Response Options | | Skip/display rules |
| 32.2 | | develop_months | | During this time period, how many **months** did [child FN] receive developmental and/or relationship-based interventions?  *Round to the nearest full month.* | | [Drop down]  1, 2, 3, 4, 5, 6, 7, 8, 9, 10, 11, 12  888=Don’t know | | Display if Q32=1 |
| 32.3 | | develop_hours | | For each active month, how many **hours per week**, on average, did [child FN] receive developmental and/or relationship-based interventions?  *For example, 1 hour of therapy every 2 weeks, would mean 0.5 hours per week.* | | [Drop down]  0=less than 1 hour  1, 2, 3, 4, 5, 6,….20  21=more than 20  888=Don’t know | | Display if Q32=1 |
| 32.4 | | develop_sessions | | During this time period, how did [child FN] receive developmental and/or relationship-based interventions?  *Check all that apply or “Don’t know.”* | | [multiselect checkboxes]  1=Individual sessions  2=Group sessions  3=777  888=Don’t know | | Display if Q32=1 |
| 32.5 | | develop_current | | Is [child FN] still receiving developmental and/or relationship-based interventions? | | [single response]  1=Yes  0=No  888=Don’t know | | Display if Q32=1 |
| 32.6 | | develop_end | | Which of the following best describes why the developmental and/or relationship-based interventions ended? | | [single response]  1=Scheduled to end after a certain time period or number of sessions  2=Not satisfied with progress/outcomes  3=Not satisfied with program/provider  4=Program/provider no longer available  5=Too expensive  6=Too burdensome  7=Child refused to go or participate  8=Child no longer needed  9=Switched to another option  777=Other  888=Don’t know | | Display if Q32.5=0 |
| Item/Q # | | Variable name | | Question Text | | Question Type and Response Options | | Skip/display rules |
| Section 3: Page 3 | | | | **Speech and Language therapy** (SLT) focuses on improving the understanding and expression of words to support communication. | | | | |
| 33 | | SLT_yn | | In the past 12 months, has [child FN] received any speech and language therapy? | | [single response]  1=Yes  0=No  888=Don’t know | |  |
| 33.1 | | SLT_location | | In the past 12 months, where did [child FN] receive speech and language therapy?  *Check all that apply or “Don’t know.”* | | [multiselect checkbox]  1=Home  2=Public school or pre-school program  3=Private school or pre-school program  4=Outpatient medical/autism clinic  5=Private therapist/clinician office  6=Daycare or before/after-school program  7=Camp  777=Other  888=Don’t know | | Display if Q33=1 |
| 33.2 | | SLT_months | | During this time period, how many **months** did [child FN] receive speech and language therapy? *Round to the nearest full month.* | | [Drop down]  1, 2, 3, 4, 5, 6, 7, 8, 9, 10, 11, 12  888=Don’t know | | Display if Q33=1 |
| 33.3 | | SLT_hours | | For each active month, how many **hours per week**, on average, did [child FN] receive speech and language therapy?  *For example, 1 hour of therapy every 2 weeks, would mean 0.5 hours per week.* | | [[Drop down]  0=less than 1 hour  1, 2, 3, 4, 5, 6,….20  21=more than 20  888=Don’t know | | Display if Q33=1 |
| 33.4 | | SLT_sessions | | During this time period, how did [child FN] receive speech and language therapy?  *Check all that apply or “Don’t know.”* | | [multiselect checkboxes]  1=Individual sessions  2=Group sessions  777=Other  888=Don’t know | | Display if Q33=1 |
| 33.5 | | SLT_current | | Is [child FN] still receiving speech and language therapy? | | [single response]  1=Yes  0=No  888=Don’t know | | Display if Q33=1 |
| Item/Q # | | Variable name | | Question Text | | Question Type and Response Options | | Skip/display rules |
| 33.6 | | SLT_end | | Which of the following best describes why the speech and language therapy ended? | | [single response]  1=Scheduled to end after a certain time period or number of sessions  2=Not satisfied with progress/outcomes  3=Not satisfied with program/provider  4=Program/provider no longer available  5=Too expensive  6=Too burdensome  7=Child refused to go or participate  8=Child no longer needed  9=Switched to another option  777=Other  888=Don’t know | | Display if Q33.5=0 |
| Section 3: page 4 | | | | **Occupational Therapy** is treatment to promote independent functioning in all aspects of daily life. The therapy focuses on improving motor skills, coordination, and ability to adapt to the surrounding environment to improve daily life skills, such as getting dressed, grooming, writing, playing, etc. | | | | |
| 34 | | OT_yn | | In the past 12 months, has [child FN] received any occupational therapy? | | [single response]  1=Yes  0=No  888=Don’t know | |  |
| 34.1 | | OT_location | | In the past 12 months, where did [child FN] receive occupational therapy?  *Check all that apply or “Don’t know.”* | | [multiselect checkbox]  1=Home  2=Public school or pre-school program  3=Private school or pre-school program  4=Outpatient medical/autism clinic  5=Private therapist/clinician office  6=Daycare or before/after-school program  7=Camp  777=Other  888=Don’t know | | Display if Q34=1 |
| 34.2 | | OT_months | | During this time period, how many **months** did [child FN] receive occupational therapy?  *Round to the nearest full month.* | | [Drop down]  1, 2, 3, 4, 5, 6, 7, 8, 9, 10, 11, 12  888=Don’t know | | Display if Q34=1 |
| Item/Q # | | Variable name | | Question Text | | Question Type and Response Options | | Skip/display rules |
| 34.3 | | OT_hours | | For each active month, how many **hours per week**, on average, did [child FN] receive occupational therapy?  *For example, 1 hour of therapy every 2 weeks, would mean 0.5 hours per week.* | | [Drop down]  0=less than 1 hour  1, 2, 3, 4, 5, 6,….20  21=more than 20  888=Don’t know | | Display if Q34=1 |
| 34.4 | | OT_sessions | | During this time period, how did [child FN] receive occupational therapy?  *Check all that apply or “Don’t know.”* | | [multiselect checkboxes]  1=Individual sessions  2=Group sessions  777=Other  888=Don’t know | | Display if Q34=1 |
| 34.5 | | OT_current | | Is [child FN] still receiving occupational therapy? | | [single response]  1=Yes  0=No  888=Don’t know | | Display if Q34=1 |
| 34.6 | | OT_end | | Which of the following best describes why the occupational therapy ended? | | [single response]  1=Scheduled to end after a certain time period or number of sessions  2=Not satisfied with progress/outcomes  3=Not satisfied with program/provider  4=Program/provider no longer available  5=Too expensive  6=Too burdensome  7=Child refused to go or participate  8=Child no longer needed  9=Switched to another option  777=Other  888=Don’t know | | Display if Q34.5=0 |
| Item/Q # | | Variable name | | Question Text | | Question Type and Response Options | | Skip/display rules |
| Section 3: page 5 | | | | **Psychological interventions** include a variety of therapies and interventions aimed at changing an individual’s thoughts, behaviors, and actions.  *Examples include:*  ***Counseling****: Interventions based on talking to a trained professional.*  ***Psychodynamic therapies****: Interventions based on exploring an individual’s subconscious thoughts and perceptions. Includes psychoanalysis and hypnotherapy.*  ***Creative therapies****: Interventions based around creative and expressive arts. Includes art therapy, dance movement therapy, drama therapy, music therapy, and play therapy.*  ***Cognitive therapies****: Interventions based on identifying and changing an individual’s thought patterns. Includes Cognitive Behavioral Therapy and Mindfulness Training.* | | | | |
| 35 | | psych_yn | | In the past 12 months, has [child FN] received any psychological interventions? | | [single response]  1=Yes  0=No  888=Don’t know | |  |
| 35.1 | | psych_location | | In the past 12 months, where did [child FN] receive psychological interventions?  *Check all that apply or “Don’t know.”* | | [multiselect checkbox]  1=Home  2=Public school or pre-school program  3=Private school or pre-school program  4=Outpatient medical/autism clinic  5=Private therapist/clinician office  6=Daycare or before/after-school program  7=Camp  777=Other  888=Don’t know | | Display if Q35=1 |
| 35.2 | | psych_months | | During this time period, how many **months** did [child FN] receive psychological interventions?  *Round to the nearest full month.* | | [Drop down]  1, 2, 3, 4, 5, 6, 7, 8, 9, 10, 11, 12  888=Don’t know | | Display if Q35=1 |
| 35.3 | | psych_hours | | For each active month, how many **hours per week**, on average, did [child FN] receive psychological interventions?  *For example, 1 hour of therapy every 2 weeks, would mean 0.5 hours per week.* | | [Drop down]  0=less than 1 hour  1, 2, 3, 4, 5, 6,….20  21=more than 20  888=Don’t know | | Display if Q35=1 |
| Item/Q # | | Variable name | | Question Text | | Question Type and Response Options | | Skip/display rules |
| 35.4 | | psych_sessions | | During this time period, how did [child FN] receive psychological interventions?  *Check all that apply or “Don’t know.”* | | [multiselect checkboxes]  1=Individual sessions  2=Group sessions  777=Other  888=Don’t know | | Display if Q35=1 |
| 35.5 | | psych_current | | Is [child FN] still receiving psychological interventions? | | [single response]  1=Yes  0=No  888=Don’t know | | Display if Q35=1 |
| 35.6 | | psych_end | | Which of the following best describes why the psychological interventions ended? | | [single response]  1=Scheduled to end after a certain time period or number of sessions  2=Not satisfied with progress/outcomes  3=Not satisfied with program/provider  4=Program/provider no longer available  5=Too expensive  6=Too burdensome  7=Child refused to go or participate  8=Child no longer needed  9=Switched to another option  777=Other  888=Don’t know | | Display if Q35.5=0 |

| Item/Q # | Variable name | Question Text | Question Type and Response Options | Skip/display rules |
| --- | --- | --- | --- | --- |
| Section 3 Page 6 | | **Other Interventions** | | |
| 36 | other_yn | In the past 12 months, has [child FN] received any other interventions?  *Check all that apply or “Don’t know”. If no other interventions were received, check “None.”* | [multiselect checkboxes]  1=Social skills training  2=Auditory integration training/therapy (AIT)  3=Sensory integration  4=Physical therapy  5=Biomedical (for example biofeedback, special diets, vitamins)  6=Academic support (for example reading, writing, and math tutor)  7=Recreational (for example specialized summer camp)  8=Animal-assisted activities and therapies (for example therapies including dogs, horses, dolphins, etc.)  9=The Built Environment  10=Social Communication/Emotional Regulation/Transactional Support (SCERTS)  11=Fast Forward, Adaptive Physical Education (APE)  12= Structured Teaching (Training and Education of Autistic and Related Communication Handicapped Children – TEACCH)  13=Other therapy not previously mentioned  888=Don’t know  14=None |  |

| Item/Q # | Variable name | Question Text | Question Type and Response Options | Skip/display rules |
| --- | --- | --- | --- | --- |
| 36.1 | other_location | In the past 12 months, where did [child FN] receive other interventions?  *Check all that apply or “Don’t know.”* | [multiselect checkbox]  1=Home  2=Public school or pre-school program  3=Private school or pre-school program  4=Outpatient medical/autism clinic  5=Private therapist/clinician office  6=Daycare or before/after-school program  7=Camp  777=Other  888=Don’t know | Display if Q36 ≠ 888 or 14 |
| 36.2 | other_months | During this time period, how many **months** did [child FN] receive other interventions?  *Round to the nearest full month.* | [Drop down]  1, 2, 3, 4, 5, 6, 7, 8, 9, 10, 11, 12  888=Don’t know | Display if Q36 ≠ 888 or 14 |
| 36.3 | other_hours | For each active month, how many **hours per week**, on average, did [child FN] receive other interventions?  *For example, 1 hour of therapy every 2 weeks, would mean 0.5 hours per week. If several other interventions were received, please count all hours for your answer.* | [[Drop down]  0=less than 1 hour  1, 2, 3, 4, 5, 6,….20  21=more than 20  888=Don’t know | Display if Q36 ≠ 888 or 14 |
| 36.4 | other_sessions | During this time period, how did [child FN] receive other interventions?  *Check all that apply or “Don’t know.”* | [multiselect checkboxes]  1=Individual sessions  2=Group sessions  777=Other  888=Don’t know | Display if Q36 ≠ 888 or 14 |
| Item/Q # | Variable name | Question Text | Question Type and Response Options | Skip/display rules |
| Section 3: Page 7 | | **Parent/Caregiver Training Interventions**  *Examples include parent/caregiver training, parent coaching or counseling, parent support groups, etc.*  *For these questions, only consider the time you spent in training or counseling. Do not include any time spent implementing the intervention or training you received.* | | |
| 37 | parent_yn | In the past 12 months, did you participate in any parent/caregiver training interventions? | [single response]  1=Yes  0=No  888=Don’t know |  |
| 37.1 | parent_months | During this time period, how many **months** did you participate in parent/caregiver training interventions?  *Round to the nearest full month.* | [Drop down]  1, 2, 3, 4, 5, 6, 7, 8, 9, 10, 11, 12  888=Don’t know | Display if Q37=1 |
| 37.2 | parent_hours | For each active month, how many **hours per week**, on average, did you participate in parent/caregiver training interventions?  *For example, 1 hour of therapy every 2 weeks, would mean 0.5 hours per week.* | [Drop down]  0=less than 1 hour  1, 2, 3, 4, 5, 6,….20  21=more than 20  888=Don’t know | Display if Q37=1 |
| 37.3 | parent_current | Are you still participating in any parent/caregiver training interventions? | [single response]  1=Yes  0=No  888=Don’t know | Display if Q37=1 |
| 37.4 | parent_end | Which of the following best describes why the parent/caregiver training interventions ended? | [single response]  1=Scheduled to end after a certain time period or number of sessions  2=Not satisfied with progress/outcomes  3=Not satisfied with program/provider  4=Program/provider no longer available  5=Too expensive  6=Too burdensome  7=No longer wanted to participate  8=No longer needed intervention  9=Switched to another option  777=Other  888=Don’t know | Display if Q37.3=0 |
| Item/Q # | Variable name | Question Text | Question Type and Response Options | Skip/display rules |
| SECTION TITLE | | **Section 4: Access and Satisfaction** | | |
| DESCRIPTION/INSTRUCTIONS | | The following questions are about access to care for your child with ASD and satisfaction with the care received. | | |
| 38 | problems_yn | In the past 12 months, did you have problems getting care for [child’s FN]’s ASD? | [single response]  1=Yes  0=No  888=Don’t know |  |
| 38.1 | problems | What problems have you had in getting care for [child FN]’s ASD? *Please check all that apply.* | [multi-select]  1=Intervention not available in my area  2=No transportation or problems with transportation  3=Inconvenient times  4=Waiting list for intervention  5=Health care provider did not know how to treat  6=Dissatisfaction with provider  7=Did not know where to go  8=Cost too much  9=No insurance  10=Health plan doesn’t cover intervention  11=No referral  12=Provider does not accept insurance  13=Other | Display if Q38=1 |
| 39 | parent_role | In the past 12 months, what has been your role in arranging care for [child FN]’s ASD?  *Please check all that apply.* | [multi-select]  1=Scheduled appointments  2=Attended appointments  3=Collected referrals  4=Coordinated care with multiple providers  5=Reported progress to providers  6=Discussed future needs/care  7=Other |  |
| Item/Q # | Variable name | Question Text | Question Type and Response Options | Skip/display rules |
| 40 | coordinator | In the past 12 months, who was the main coordinator of care for [Child FN]’s ASD? | [single response]  1=Primary health care provider  2= Other professional (such as therapist or counselor)  3=You (the parent/caregiver)  4=Other (non-professional)  888=Don’t know |  |
| 41 | coord_satisfaction | How satisfied are you with the **coordination of care** for [Child FN]’s ASD? | [single response]  1=Extremely satisfied  2=Very satisfied  3=Slightly satisfied  4=Neither satisfied nor dissatisfied  5=Slightly dissatisfied  6=Very dissatisfied  7=Extremely dissatisfied |  |
| 42 | care_satisfaction | How satisfied are you with the **care** for [child FN]’s ASD? | [single response]  1=Extremely satisfied  2=Very satisfied  3=Slightly satisfied  4=Neither satisfied nor dissatisfied  5=Slightly dissatisfied  6=Very dissatisfied  7=Extremely dissatisfied |  |
| 43 | care_benefits | In the past 12 months, in your opinion, how much has the care helped [Child FN]’s ASD? | [single response]  1=Very much  2=Much  3=Somewhat  4=Not at all  888=Don’t know |  |
| END of Survey 1 | | | | |
| Go to Autism Impact Measure (AIM): **Redacted for copyright reasons** | | | | |
